# Mammary tumour cells remodel the bone marrow vascular microenvironment to support metastasis

**DOI:** 10.1038/s41467-021-26556-6

**Published:** 2021-11-26

**Authors:** Raymond K. H. Yip, Joel S. Rimes, Bianca D. Capaldo, François Vaillant, Kellie A. Mouchemore, Bhupinder Pal, Yunshun Chen, Elliot Surgenor, Andrew J. Murphy, Robin L. Anderson, Gordon K. Smyth, Geoffrey J. Lindeman, Edwin D. Hawkins, Jane E. Visvader

**Affiliations:** 1 ACRF Cancer Biology and Stem Cells Division, The Walter and Eliza Hall Institute of Medical Research, Parkville, VIC 3052 Australia; 2 Department of Medical Biology, The University of Melbourne, Parkville, VIC 3010 Australia; 3 Inflammation Division, The Walter and Eliza Hall Institute of Medical Research, Parkville, VIC 3052 Australia; 4 School of Cancer Medicine, La Trobe University, Bundoora, VIC 3086 Australia; 5 Olivia Newton-John Cancer Research Institute, Heidelberg, VIC 3084 Australia; 6 Bioinformatics Division, The Walter and Eliza Hall Institute of Medical Research, Parkville, VIC 3052 Australia; 7 Department of Immunology, Monash University, Melbourne, VIC Australia; 8 Division of Immunometabolism, Baker Heart & Diabetes Institute, Melbourne, VIC Australia; 9 School of Mathematics and Statistics, The University of Melbourne, Parkville, VIC 3010 Australia; 10 Department of Medicine, The University of Melbourne, Parkville, VIC 3010 Australia; 11 Department of Medical Oncology and Parkville Familial Cancer Centre, The Peter MacCallum Cancer Centre and Royal Melbourne Hospital, Parkville, VIC 3050 Australia

**Keywords:** Breast cancer, Cellular imaging, Breast cancer

## Abstract

Bone marrow is a preferred metastatic site for multiple solid tumours and is associated with poor prognosis and significant morbidity. Accumulating evidence indicates that cancer cells colonise specialised niches within the bone marrow to support their long-term propagation, but the precise location and mechanisms that mediate niche interactions are unknown. Using breast cancer as a model of solid tumour metastasis to the bone marrow, we applied large-scale quantitative three-dimensional imaging to characterise temporal changes in the bone marrow microenvironment during disease progression. We show that mouse mammary tumour cells preferentially home to a pre-existing metaphyseal domain enriched for type H vessels. Metastatic lesion outgrowth rapidly remodelled the local vasculature through extensive sprouting to establish a tumour-supportive microenvironment. The evolution of this tumour microenvironment reflects direct remodelling of the vascular endothelium through tumour-derived granulocyte-colony stimulating factor (G-CSF) in a hematopoietic cell-independent manner. Therapeutic targeting of the metastatic niche by blocking G-CSF receptor inhibited pathological blood vessel remodelling and reduced bone metastasis burden. These findings elucidate a mechanism of ‘host’ microenvironment hijacking by mammary tumour cells to subvert the local microvasculature to form a specialised, pro-tumorigenic niche.

## Abstract (web summary)

The visualisation of the bone metastasis process in a spatial temporal manner is lacking. Here, the authors use three-dimensional quantitative imaging and show that mouse mammary tumour cells preferentially home to endothelial subtype type H vessels within the bone marrow and remodel this vasculature by producing granulocyte-colony stimulating factor.

## Introduction

Solid tumours frequently metastasise to bone, a process that is rarely curable. Bone metastasis is particularly common in patients with advanced breast and prostate cancers, occurring in approximately 70% and 90% of patients, respectively^1^. Tumour invasion into bone can be associated with severe bone pain, fractures, life-threatening hypercalcaemia and reduced mobility that greatly impact on the quality of life^2^. The success of disseminated tumour cells (DTCs) in establishing themselves in bone marrow (BM) is determined by intrinsic cellular characteristics as well as tumour−stromal interactions^3^.

The bone microenvironment contains a variety of hematopoietic cells and stromal cells including osteoblasts, osteoclasts, endothelial cells, and mesenchymal cells^4^. At the initial invasion stage, DTCs seed specialised regions or ‘niches’ in the marrow that are favourable for colonisation. They can engage with niche components such as osteoblasts to activate intrinsic pro-survival pathways^5^, evade immunosurveillance^6^ and receive cues from the microenvironment to either initiate lesion outgrowth or enter dormancy^7,8^. In breast cancer, the expansion of metastatic lesions is associated with the activation of bone-degrading osteoclasts that release bone-bound molecules and initiate a feedback loop to cancer cells that stimulates their proliferation^9,10^. Emerging evidence suggests that the bone vasculature mediates the homing of DTCs and acquisition of stem cell traits^11,12^, thus regulating the balance between dormant and proliferative cell states^13^. In addition, the perivascular niche has been shown to confer chemotherapeutic resistance to resident DTCs^14^. These findings highlight the complex and constantly evolving symbiosis between tumour cells and stromal components during BM colonisation. Although these interactions are critical to disease pathology, high-resolution spatiotemporal studies of solid tumour cell infiltration of the BM space are lacking. Understanding the features of metastatic niches and the drivers of niche formation is crucial for the prevention and the treatment of metastatic disease.

In this study, we used an immunocompetent mouse model of spontaneous breast cancer bone metastasis and performed three-dimensional (3D) quantitative imaging of mouse BM to map the location of metastatic niches and to analyse tumour−stromal interactions in situ during early colonisation and expansion. In the initial dissemination phase, invading mammary tumour cells were demonstrated to home preferentially to a distinct endosteal vascular subtype, termed type H vessels. Formation of overt metastases occurred concurrently with profound remodelling of the local vascular architecture, predominantly mediated by tumour-derived granulocyte-colony stimulating factor (G-CSF). Blockade of this microenvironment signal attenuated BM vessel remodelling and bone metastasis progression. These data suggest that solid tumour metastases are distinct cellular entities that remodel the host microenvironment to form specialised niches to support their expansion.

## Results

### Mammary DTCs predominantly localise adjacent to type H vessels within bone

**Fig. 1:**
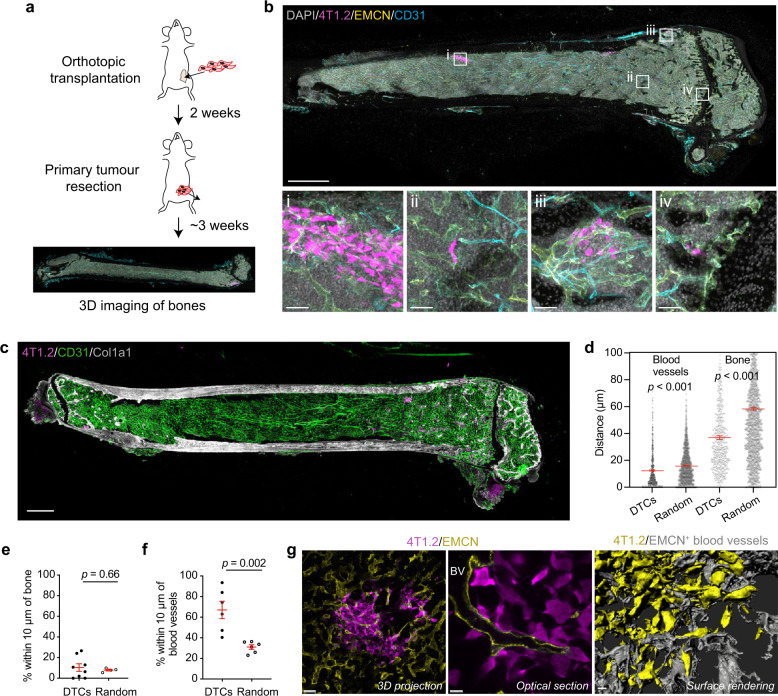
Metastatic mammary cancer cells selectively home to a BM vascular niche. **a** Experimental workflow: 4T1.2-RFP cells were orthotopically transplanted into BALB/c mice. Primary tumours were resected 2 weeks later, bones collected at ethical endpoint, cryopreserved and then immunolabelled. **b** Top: tile scan covering the entire femoral slice with a Z-depth of ~100 µm. Bones were stained for DAPI (grey), 4T1.2 disseminated tumour cells (DTCs; magenta), Endomucin (EMCN; yellow) and CD31 (cyan). Bottom: four examples (corresponding to (i)−(iv) from the top image) of 4T1.2 DTCs either as solitary or clustered cells (*n* = 11 mice). Scale bars: 1 mm (top), 50 µm (bottom). **c** 3D image covering the entire femoral BM immunostained for 4T1.2 DTCs (magenta), CD31 (green) and Col1a1 (grey) (*n* = 11 mice). Scale bar, 1 mm. **d** Distance to the nearest blood vessel or bone surface for individual 4T1.2 DTC or simulated random spot. A total of 1426 DTCs were analysed from 11 different mice. *P* values, two-tailed unpaired *t* tests. **e**, **f** Percentages of 4T1.2 DTCs and random spots within 10 µm of a bone surface (**e**) or blood vessel (**f**). For (**e**) and (**f**), a total of 655 DTCs and 758 DTCs were analysed in 8 and 6 mice, respectively. *P* values, two-tailed unpaired *t* tests. **g** Optical section and surface rendering from 3D image of a 4T1.2 micrometastasis (magenta) in bone marrow immunostained for EMCN (yellow). In surface rendering, 4T1.2 cells and blood vessels were pseudo-coloured to yellow and grey, respectively (*n* = 11 mice). BV blood vessel. Scale bars: 50 µm (left), 10 µm (middle and right). All data reflect mean ± s.e.m. Source data are provided as a Source Data file.

To determine whether solid tumour cells preferentially metastasise to a specific niche in the BM, we orthotopically implanted highly metastatic 4T1.2 mammary cancer cells (BALB/c-derived) expressing red fluorescent protein (RFP) into BALB/c mice and monitored spontaneous bone metastasis (Fig. 1a). This mouse tumour cell line has a propensity to spontaneously metastasise from the mammary fat pad to bone, resembling metastasis observed in human breast cancer^15^. We adapted protocols previously developed to image hematopoietic stem cell niches to map the distribution of DTCs in the bone microenvironment^16,17^. Free-floating immunolabelling and optical clearing techniques were implemented to enhance antibody penetration and detection sensitivity. After antibody staining of thick whole bone sections, the specimens were cleared for combined confocal and multiphoton microscopy to acquire high-resolution, tiled Z-stacks throughout the marrow to a depth of ~100 µm. Using this approach, individual DTCs or clusters could be accurately identified and their position measured relative to other components in the bone marrow, such as blood vessels identified by expression of Endomucin (EMCN) and CD31 (or PECAM1) (Fig. 1b). Moreover, open blood vessel lumens were visible and tissue architecture appeared to be preserved (Supplementary Movie 1).

To catalogue the different stages of bone metastasis, we collected tumour-burdened mice at different time points following orthotopic transplantation (between 14 and 28 days). The majority of DTCs resided in close proximity to the growth plate, regardless of the stage of metastasis (Supplementary Fig. 1a, b). The trabecular area beneath the growth plate is highly vascularised and is a hotspot of bone remodelling^18^. To determine if DTCs rely on specific cell types for homing to the BM, we measured the distance between individual tumour cells and either bone or vascular components within the BM (Fig. 1c). DTCs were significantly more likely to be found near blood vessels and bone surfaces compared to random generated spots (Fig. 1d). Furthermore, when location was categorised within 10 µm of the vasculature and bone structures, we found that the majority of DTCs were preferentially localised near blood vessels (vasculature: 67.1 ± 8.5% DTCs; bone surface: 7.3 ± 4.2% of DTCs) (Fig. 1e, f). To further visualise the perivascular localisation of DTCs, we performed surface rendering on high-resolution 3D data sets. Intimate cell−cell contact between DTCs and the vascular endothelium was evident (Fig. 1g and Supplementary Movie 2), suggesting that the vascular niche primarily supports BM infiltration.

**Fig. 2:**
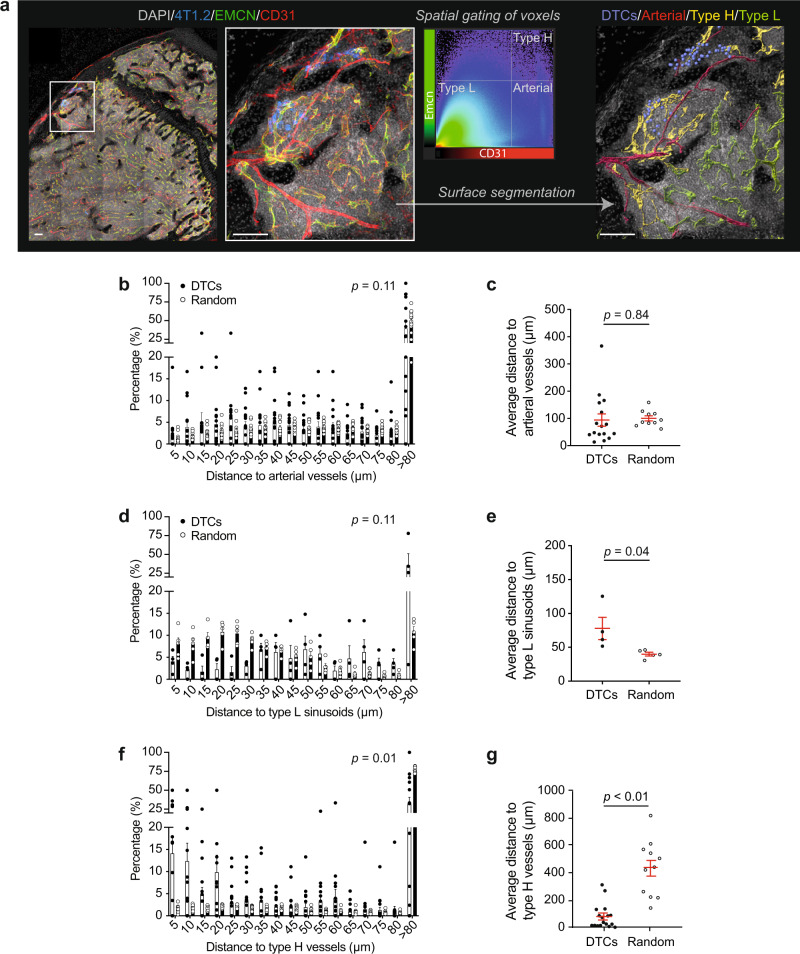
A specialised vascular niche for mammary cancer cells during bone colonisation. **a** 3D confocal image of DTCs (blue) in bone marrow. Bones were stained for DAPI (grey), EMCN (green) and CD31 (red). The image was transformed into a density plot that displayed voxel intensities in the CD31 and EMCN channels. Spatial gates were drawn to split the data set into phenotypically distinct components for volumetric reconstructions. Arterial vessels were defined by CD31^hi^EMCN^–^ (red); Type H capillaries by CD31^hi^EMCN^hi^ (yellow); Type L sinusoids by CD31^lo^EMCN^lo^ (green) (*n* = 4 mice). Scale bars: 100 µm. **b**, **d**, **f** Distance to the nearest arterial vessel (**b**), type L sinusoid (**d**) and type H vessel (**f**) of individual DTCs and random spots were binned in histogram format. **c**, **e**, **g** Bar charts showing the averages of distances to vessel subtypes. Individual dots are from metastases from different mice. A total of 1256 DTCs (**b**, **c**), 299 DTCs (**d**, **e**) and 1052 DTCs (**f**, **g**) were analysed in 16 (**b**, **c**), 4 (**d**, **e**) and 17 (**f**, **g**) mice, respectively. *P* values in (**b**), (**d**), (**f**) by two-sided Kolmogorov−Smirnov analysis, and in (**c**), (**e**), (**g**) by two-tailed unpaired *t* tests. All data reflect mean ± s.e.m. Source data are provided as a Source Data file.

The BM vasculature is hierarchically organised and consists of multiple vessel subtypes with distinct functional properties^19^. To characterise vessels that preferentially interact with DTCs, voxel colocalization analysis was performed on 3D images to discriminate and create digital surfaces for arterial vessels (CD31^hi^EMCN^–^), type H capillaries (CD31^hi^EMCN^hi^), and type L sinusoids (CD31^lo^EMCN^lo^), and spots that represent DTCs (Fig. 2a). Following this labelling scheme, we found that DTCs were not significantly enriched near arterioles in comparison to random spots, with less than 10% found within 10 µm of an arterial vessel (Fig. 2b, c and Supplementary Fig. 1c). In contrast, the distribution of DTCs relative to type L sinusoids was slightly skewed, with most tumour cells localised further from these sinusoids (Fig. 2d, e and Supplementary Fig. 1d). Our observation that a large proportion of random spots was more than 30 µm from the closest sinusoid differs from earlier reports^20,21^ and suggests that sinusoidal density and distribution were altered even at the early stages of metastatic spread. Strikingly, DTCs were found to be closely associated with type H vessels: 24.9 ± 6.2% of DTCs were located within 10 µm of a type H vessel compared to 3.8 ± 1.5% for simulated data (Fig. 2f, g and Supplementary Fig. 1e). Similar observations were noted for the murine 67NR and the human MDA-MB-231 tumour cell lines (Supplementary Fig. 1f). Thus, mammary cancer cells preferentially localise to a distinct vascular niche comprised of type H vessels.

Tumours have been reported to educate distant organs through secreted factors to form a tumour-receptive microenvironment before the arrival of DTCs (termed the pre-metastatic niche). To address the possibility that type H vessels were influenced by primary mammary 4T1.2 tumours to form a pre-metastatic niche, we introduced 4T1.2 cells directly into the arterial circulation via the intracardiac route, thus bypassing primary tumorigenesis. As seen in the primary orthotopic model, mammary DTCs were selectively found adjacent to type H vessels, indicating that they home to a pre-existing and dissemination-permissive vascular niche (Supplementary Fig. 1g, h).

### Breast cancer bone metastasis drives remodelling of the local vasculature

**Fig. 3:**
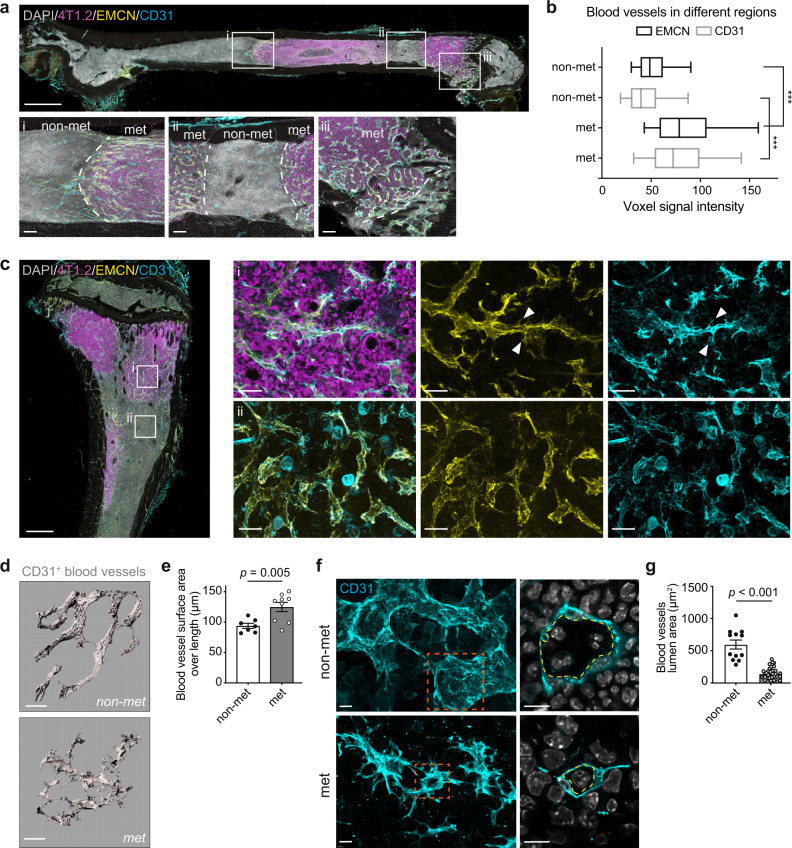
Metastatic mammary cancer cells remodel the bone marrow vasculature upon expansion. **a** Tile scan (top) and enlarged (bottom) 3D images of femoral BM stained for DAPI (grey), 4T1.2 cells (magenta), EMCN (yellow) and CD31 (cyan) reveal vessel remodelling in tumour-infiltrated area (met) but not in the adjacent tumour-free area (non-met) (*n* = 5 mice). Scale bars, 1 mm (top), 100 µm (bottom). **b** Box and whisker plots of mean vascular CD31 and EMCN voxel intensity of individual vessels in non-met and met areas from *n* = 5 mice. Centre line; median; box limits, from the 25th to 75th percentiles; whiskers, from the 5th to 95th percentiles. ***P < 0.0001 by two-tailed unpaired *t* test. **c** Left: 3D image of femoral BM stained for DAPI (grey), 4T1.2 cells (magenta), EMCN (yellow) and CD31 (cyan). Right: zoomed-in images of met and non-met area (corresponding to (i) and (ii) from the left panel, respectively) (*n* = 7 mice). Arrowheads indicate EMCN^+^CD31^+^ vascular sprouts. Scale bars: 500 µm (overview), 50 µm (enlargement). **d** Digital reconstructions of CD31-positive blood vessels in non-met and met areas. Scale bar, 50 µm. **e** Measurements of endothelial surface area over vessel length from (**d**). *n* = 7 (non-met) and 9 mice (met). *P* value, two-tailed unpaired *t* tests. **f** 3D images of CD31-positive blood vessels (cyan) in non-met and met area. Right panel shows optical section from the selected area in left panel. Yellow lines demarcate vessel lumen. Scale bars: 20 µm. **g** Lumen area from (**f**). Individual dots are from one vessel. *n* = 5 (non-met) and 10 mice (met). *P* value, two-tailed unpaired *t* tests. All data reflect mean ± s.e.m. Source data are provided as a Source Data file.

High-resolution 3D imaging was next applied to study the interplay between tumour and stroma during metastatic lesion outgrowth. Intracardial injection of 4T1.2 cells was implemented to achieve efficient bone colonisation given that homing to type H vessels occurred irrespective of the route of transplantation. 3D confocal imaging revealed profound changes in the BM vasculature within macroscopic bone lesions (Fig. 3a). Blood vessels in heavily infiltrated areas (met) were tortuous and severely disorganised compared to the adjacent tumour-free endothelium (non-met) (Fig. 3a), concomitant with a significant reduction in blood vessel coverage within met areas (Supplementary Fig. 2a). Metastasis-associated vessels showed a dramatic increase in CD31 and EMCN signal intensity (Fig. 3b). This immunophenotypic change was spatially restricted to macroscopic lesions as the neighbouring sinusoidal vessels maintained low immunoreactivity for CD31 and EMCN (Fig. 3a(i)−(iii), b). Despite acquiring a CD31^hi^EMCN^hi^ expression signature, metastasis-associated vessels lacked features characteristic of metaphyseal type H vessels. For example, they were not associated with Osx^+^ osteoprogenitors or covered by PDGFRβ^+^ pericytes (Supplementary Fig. 2b, c). Since most breast cancer bone metastases generate osteolytic bone lesions, we expanded our analysis of the metastatic niche to include osteoblastic cells (CFP^+^ cells in Col2.3-CFP reporter mice backcrossed onto a BALB/c background). In parallel with vessel remodelling, 3D imaging of bone macrometastases in Col2.3-CFP mice revealed dispersion of CFP^+^ cells throughout the lesion as opposed to their normal localisation at the bone surface (Supplementary Fig. 2d). These findings suggest that mammary DTCs focally transform vascular and osteoblastic niches during lesion expansion.

The remodelling of bone marrow niches has been characterised in multiple haematological malignancies^22–26^; however, less is known in the context of solid tumour bone metastasis. Through enhanced resolution imaging, we could resolve unappreciated subcellular features of the intralesional vasculature. Morphologically, blood vessels in metastases appeared unusually barbed and presented CD31^+^EMCN^+^ cytoplasmic protrusions whereas adjacent endothelium was unaffected (Fig. 3c). 3D reconstructions of these vessels within metastases revealed extensive sprout formation along the endothelium, measured as an increase in endothelial surface area over vessel length (Fig. 3d, e). In addition, optical slicing of metastasis-associated endothelium showed that the mean vascular lumen area was reduced (Fig. 3f, g), a pathologic phenotype previously described in tumour angiogenesis to result from compressive forces exerted on vessels by expanding tumour cells^27^. Of note, CD31^hi^EMCN^–^ arterial blood vessels were unaffected, likely because they are ensheathed by supportive αSMA^+^ pericytes (Supplementary Fig. 2e, f). The absence of Ki67 and cleaved caspase 3 (CC3) signals in metastasis-associated vessels indicated that the remodelling process was not driven by abnormal proliferation or apoptosis of ECs (Supplementary Fig. 2g, h).

### Molecular interactions between mammary tumour cells and bone ECs

**Fig. 4:**
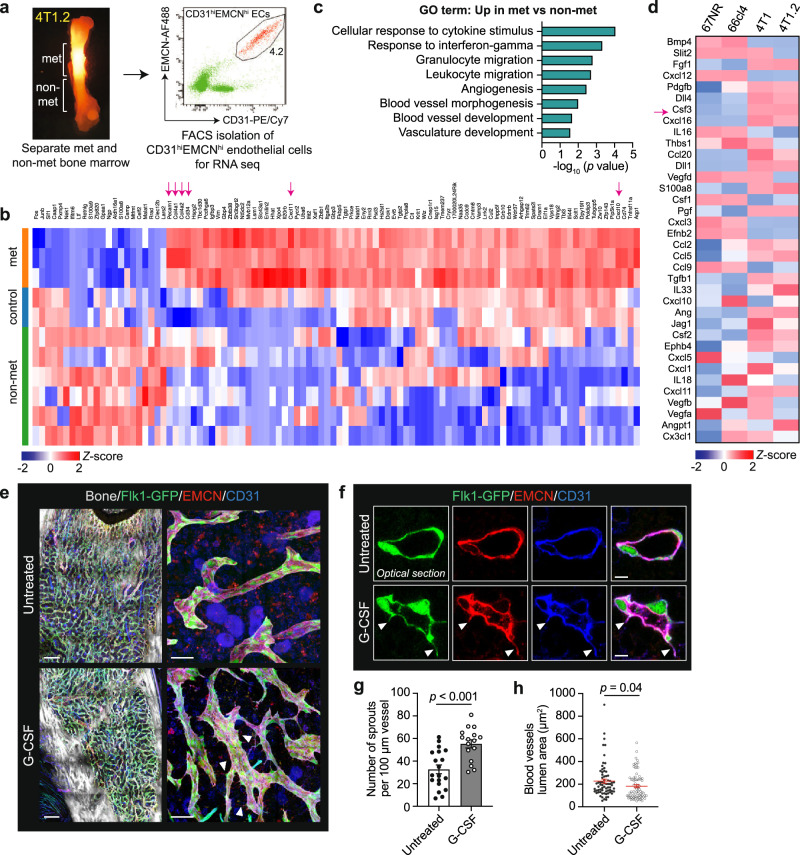
G-CSF induces vascular remodelling in bone marrow. **a** Tumour-infiltrated (met) and adjacent tumour-free (non-met) areas were microdissected and sorted to isolate CD31^hi^EMCN^hi^ endothelial cells (ECs) for RNA-seq. Bones from age-matched, healthy BALB/c mice were collected as controls. Representative FACS plot showing the gating profile (see Supplementary Fig. 3a). **b** Expression levels of top differentially expressed genes in CD31^hi^EMCN^hi^ ECs retrieved from met and non-met areas and control bones. The heat map shows the mean-centred *z*-score (*n* = 3 (met), 2 (control) and 6 (non-met) samples). Arrows designate angiogenesis or inflammatory response-related genes. **c** Gene ontology analysis conducted on the transcriptomes of CD31^hi^EMCN^hi^ ECs isolated from met and non-met areas. **d** Heat map shows the mean-centred *z*-score of selected genes in angiogenesis and the inflammatory response across four isogenic murine mammary tumour lines. Genes were ranked by false discovery rate (FDR; *n* = 2 replicates per cell line). **e** Left: multiphoton 3D images of femoral BM of *Flk1-GFP* (green) reporter mice following G-CSF treatment. Bones were immunostained for EMCN (red) and CD31 (blue). Bone collagen was defined by second harmonic signal (grey). Right: high-magnification images of marrow endothelium in the corresponding mice (*n* = 3 mice per group). Arrowheads mark protruding vascular sprouts. See also Supplementary Movies 4 and 5. Scale bars, 200 µm (overviews), 20 µm (enlargements). **f** Optical sections of marrow endothelium from (**e**). Arrowheads mark protruding vascular sprouts. Scale bars, 5 µm. **g**, **h** Number of endothelial sprouts per 100 µm of vessel length (**g**) and vessel lumen area (**h**) from (**e**). Individual dots are from one vessel. *n* = 3 mice per group. *P* values, two-tailed unpaired *t* tests. All data reflect mean ± s.e.m. Source data are provided as a Source Data file.

To delineate molecular pathways driving vascular remodelling within the metastatic niche, we microdissected met and non-met areas from 4T1.2-burdened bones and used flow cytometry to enrich for CD31^hi^EMCN^hi^ bone-metastasis-associated ECs (BmECs) for RNA-seq analysis (Fig. 4a and Supplementary Fig. 3a). A comparison of the transcriptomes of BmECs and the same EC subset retrieved from the adjacent non-met area or healthy bone identified a distinctive gene signature for BmECs (Fig. 4b). Gene ontology enrichment analysis highlighted several altered processes primarily associated with angiogenesis, blood vessel morphogenesis and immune cell migration (Fig. 4c). The expression of vascular basement membrane markers, *Col4a1* and *Col4a2*, increased dramatically upon tumour infiltration. We also noted significant upregulation of the interferon-inducible chemokines *Cxcl10* and *Cxcl11*, which are strong chemo-attractants for inflammatory cells^28^ (Fig. 4b). Interestingly, the most upregulated gene in ECs retrieved from non-met regions was *S100a8*, a critical pro-inflammatory mediator that can promote neutrophil recruitment and neaoangiogenesis^29,30^. These data suggest that solid tumour expansion creates a localised tumour microenvironment in BM with molecular changes associated with the hallmarks of inflammation.

To explore how remodelling of the tumour microenvironment might be driven by tumour cells, we studied the transcriptomes of 4T1 and 4T1.2 cells and their sibling cell lines, 66cl4 and 67NR, which harbour low metastatic potential compared to 4T1 and 4T1.2 cells. Analysis of top upregulated differentially expressed (DE) genes in 4T1/4T1.2 cells, with emphasis on growth factors and chemokines that are associated with inflammatory responses and angiogenesis, revealed a number of candidates. These included the known modulators of angiogenesis, *Fgf1*^31^*, Pdgfb*^32^ and *Dll4*^33^ as well as multiple chemokines (e.g. *Cxcl16, Cxcl11, Ccl5*). In line with previous RNA expression and ELISA assays^34^, *Csf3*, which encodes the granulocyte colony stimulating factor (G-CSF), was significantly upregulated in 4T1.2 cells relative to 66cl4 and 67NR cells (Fig. 4d). Increased G-CSF production has been implicated in breast cancer pulmonary metastasis through the activation of neutrophils in forming the pre-metastatic niche^35,36^, but the physiological effect of this cytokine on the BM endothelium remains unclear.

### Tumour-derived G-CSF remodels BM vasculature to create a pro-metastatic niche

To investigate the role of G-CSF in BM vessel remodelling, we injected naïve *Flk1-GFP* transgenic mice, which express GFP in ECs, with Filgrastim (recombinant human G-CSF) and performed large-scale 3D multiphoton imaging on optically cleared femoral cavities, allowing visualisation ranging from organ-wide to subcellular levels (Supplementary Fig. 3b and Supplementary Movie 3). While G-CSF treatment did not alter the density of BM vasculature (Supplementary Fig. 3c), high-resolution images uncovered the presence of numerous thin, elongated projections along the marrow endothelium in G-CSF-treated mice (Fig. 4e and Supplementary Movies 4 and 5). These cytoplasmic processes were morphologically similar to those within the bone metastatic niche; they emanated from the Flk1-GFP^+^ vascular basement membrane into the extravascular space and expressed high levels of EMCN and CD31 (Fig. 4f). Large tile scans of bones along the longitudinal axis revealed vessel remodelling occurred on a global scale rather than locally perturbing BM architecture (Supplementary Fig. 3d). Owing to the heterogeneous trajectories of endothelial projections, we focused on transversal sections of BM vessels and measured their morphological traits. Notably, the endothelium of G-CSF-treated mice had a significantly higher density of vascular sprouts and a marked reduction in intravascular lumen area (Fig. 4g, h), demonstrating that exogenous G-CSF administration remodels the micro-architecture of BM vessels in vivo.

Within the bone metastatic niche, G-CSF may be produced by tumour cells or host stroma in response to cancer colonisation. To distinguish the two possibilities, we first compared the effects of the G-CSF-secreting 4T1.2 cell line versus the non-producing 67NR cell line^34^ on the BM endothelium following intracardial inoculation. In contrast to the 4T1.2 model, blood vessels in 67NR-induced bone lesions did not develop endothelial sprouts despite a tortuous appearance owing to compression by surrounding tumour cells (Supplementary Fig. 3e). We next examined bone macrometastases derived from human MDA-MB-231 breast cancer cells, which secrete human G-CSF^35^ at levels approximately 7-fold lower than that produced by 4T1 cells. 3D imaging revealed relatively normal intralesional vasculature with little evidence of sprouting and vessel destruction, albeit a slightly distorted vessel network (Supplementary Fig. 3f). These findings imply that sprout formation is not a consequence of physical insult on the vessels or a bystander effect of crowded tumour colonisation.

**Fig. 5:**
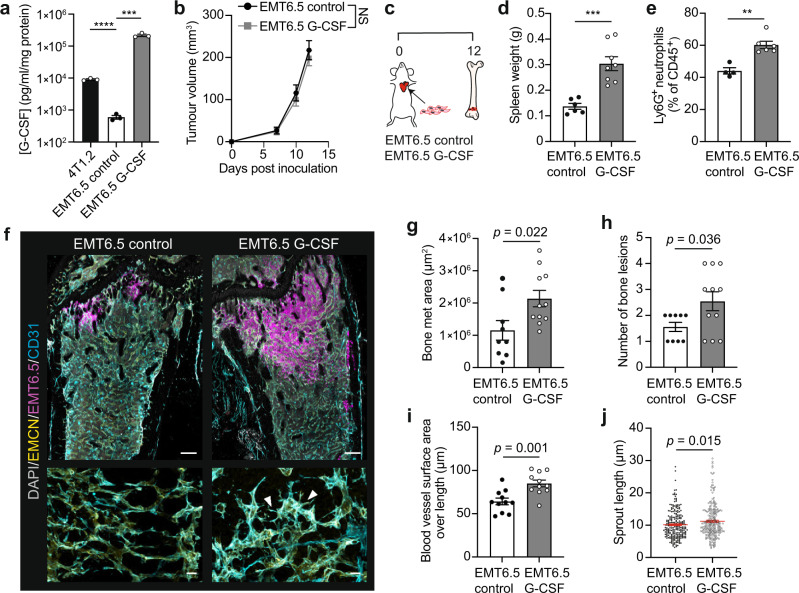
Tumoral G-CSF drives BM vascular remodelling and enhances bone metastasis development. **a** ELISA for G-CSF in cell media of 4T1.2, EMT6.5 control and EMT6.5 G-CSF cells. *n* = 3 per cell line. ***P = 0.0004, ****P < 0.0001 by two-tailed unpaired *t* tests. **b** Orthotopic tumour growth of EMT6.5 control (*n* = 10 mice) and EMT6.5 G-CSF cells (*n* = 8 mice). *P* value, two-way repeated measures ANOVA (days 0−12). **c** Experimental plan to test the effect of cancer cell-secreted G-CSF on bone metastasis**. d**, **e** Spleen weight (**d**) and quantification of circulating Ly6G^+^ neutrophils (**e**) at experimental endpoint. For (**d**), *n* = 6 (control) and 8 mice (G-CSF). For (**e**), *n* = 4 (control) and 6 mice (G-CSF). **P = 0.001, ***P = 0.0003 by two-tailed unpaired *t* tests. **f** 3D images (top) and enlarged micrographs (bottom) of femur from mice injected with control or G-CSF-expressing EMT6.5 cells. Bones were stained for DAPI (grey), EMT6.5 cells (magenta), EMCN (yellow) and CD31 (cyan) (*n* = 9 mice per cell line). Arrowheads indicate endothelial sprouts. Scale bars: 200 µm (overviews), 20 µm (enlargements). **g**, **h** Measurement of lesion area (**g**) and number (**h**) from (**f**). *n* = 9 (control) and 11 mice (G-CSF). *P* values, two-tailed unpaired *t* tests. **i**, **j** Quantification of surface area over vessel length (**i**) and sprout length (**j**) of blood vessels in macroscopic bone lesions from mice harbouring control and G-CSF-expressing EMT6.5 cells. Individual dots are from one vessel (**i**) or sprout (**j**). For (**i**), *n* = 4 (control) and 5 mice (G-CSF). For (**j**), *n* = 3 mice per group. *P* values, two-tailed unpaired *t* tests. All data reflect mean ± s.e.m. NS not significant. Source data are provided as a Source Data file.

To evaluate whether tumour cell-intrinsic G-CSF production drives vessel remodelling, we expressed *Csf3* in EMT6.5 mammary tumour cells (Fig. 5a). Of note, this is a distinct, syngeneic model of breast cancer metastasis and these cells produce little G-CSF in comparison to 4T1.2. (Fig. 5a). Forced expression of *Csf3* had no impact on primary tumour growth (Fig. 5b). As expected, intracardiac inoculation of *Csf3*-overexpressing EMT6.5 cells into immunocompetent BALB/c mice led to splenomegaly and an increase of host neutrophils in peripheral blood (Fig. 5c–e). *Csf3*-EMT6.5 cells, but not control cells, caused profound sprouting of BM endothelium in macroscopic lesions and exacerbated bone metastasis, reflected by an increase in bone lesion number and size (Fig. 5f–j). Taken together, these in vivo results highlight the ability of tumour-secreted G-CSF to remodel host vascular niches and promote bone metastatic growth of mammary tumours.

### G-CSF stimulates BM endothelium through a non-hematopoietic mechanism

**Fig. 6:**
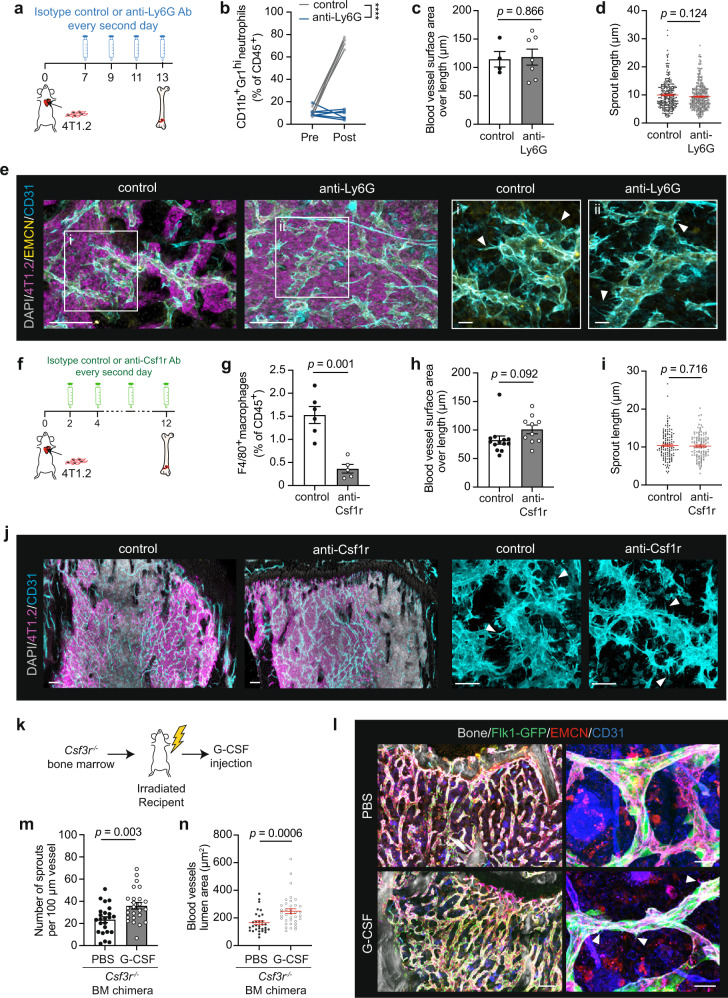
G-CSF-induced vessel remodelling is independent of hematopoietic cells. **a** Diagram showing the experimental plan in (**b**–**e**). **b** FACS analysis of circulating neutrophils before and after treatment. *n* = 5 (control) and 6 mice (anti-Ly6G). ****P < 0.0001. **c**, **d** Quantification of surface area over vessel length (**c**) and sprout length (**d**) of blood vessels from (**e**). Individual dots are from one vessel (**c**) or sprout (**d**). For (**c**), *n* = 3 (control) and 5 mice (anti-Ly6G). For (**d**), *n* = 3 mice per group. **e** Tile scan (left) and magnified images (right) of bone lesions from mice that received isotype control or anti-Ly6G antibody. Bones were stained for DAPI (grey), 4T1.2 cells (magenta), EMCN (yellow) and CD31 (cyan) (*n* = 5 mice per group). Arrowheads mark protruding vascular sprouts. Scale bars: 50 µm (left), 30 µm (right). **f** Diagram showing the experimental plan in (**g**−**j**). **g** Quantification of bone marrow F4/80^+^ macrophages at experimental endpoint. *n* = 6 (control) and 5 mice (anti-Csf1r). **h**, **i** Quantif**i**cation of surface area over vessel length (**h**) and sprout length (**i**) of blood vessels from (**j**). Individual dots are from one vessel (**h**) or sprout (**i**). For (**h**), *n* = 6 (control) and 5 mice for *(*anti-Csf1r). For (**i**), *n* = 3 mice per group. **j** Tile scan (left) and magnified images (right) of bone lesions from mice that received isotype control or anti-Csf1r antibody. Bones were stained for DAPI (grey), 4T1.2 cells (magenta) and CD31 (cyan) (*n* = 5 mice per group). Arrowheads point to protruding vascular sprouts. Scale bars: 200 µm (overview), 20 µm (enlargements). **k** Diagram showing the experimental plan in (**l**−**n**). **l** Multiphoton 3D images of femur from G-CSF-treated and control *Flk1-GFP* (green) mice reconstituted with *G-CSFR*-deficient haematopoiesis. Bones were immunostained for EMCN (red) and CD31 (blue). Bone collagen was defined by second harmonic signal (*n* = 3 mice per group). Arrowheads mark protruding vascular sprouts. Scale bars: 100 µm (overviews), 20 µm (enlargements). **m**, **n** Number of endothelial sprouts per 100 µm of vessel length (**m**) and vessel lumen area (**n**) from (**l**). Individual dots are from one vessel (*n* = 3 mice per group). *P* values in (**b**) by repeated measure two-way ANOVA, and in (**c**), (**d**), (**g**), (**h**), (**i**), (**m**), (**n**) by two-tailed unpaired *t* test. All data reflect mean ± s.e.m. Source data are provided as a Source Data file.

G-CSF binds and signals exclusively through the G-CSF receptor (G-CSFR). *Csf3r*, which encodes G-CSFR, is predominantly expressed on neutrophils, but can be found at low levels on some myeloid derivatives, a subset of lymphocytes, and non-hematopoietic cells such as endothelial cells^37^. We therefore investigated whether tumour-derived G-CSF acts through hematopoietic intermediates to influence vessel biology using a range of cell depletion approaches. Initially, we examined the role of lymphoid and NK cell lineages. We inoculated G-CSF-producing 4T1.2 cells into immunodeficient NSG mice (NOD/SCID/IL2Rγc^−/−^) that lack functional B, T and NK cells but retain myeloid cells. The BM of NSG mice showed vessel sprouting in areas heavily infiltrated with tumour cells, demonstrating that vessel remodelling is not dependent on these cell types (Supplementary Fig. 4a). As neutrophils are the principal cell type that respond to G-CSF activity, we next evaluated their role in vascular remodelling. We introduced 4T1.2 cells into immunocompetent mice by intracardiac injection, allowed 1 week for bone lesions to develop and then treated metastasis-bearing mice with an anti-Ly6G antibody (Ab) to deplete neutrophils (Fig. 6a). As expected, anti-Ly6G Ab treatment led to a significant decrease in the frequency of CD11b^+^Gr1^hi^ neutrophils in the peripheral blood and BM without perturbing spleen weight (Fig. 6b and Supplementary Fig. 4b, c). However, 3D visualisation and quantitative analysis of the metastasis-associated vessels did not reveal a significant decrease in the frequency or length of vascular sprouts, and vessel density within the tumour-infiltrated area was unchanged (Fig. 6c–e and Supplementary Fig. 4d). In accordance, the incidence and severity of bone metastases were unaffected (Supplementary Fig. 4e, f). Since the remodelled vasculature in the metastatic niche was not rescued by neutrophil depletion, we performed in situ staining to detect neutrophils in bone lesions of untreated mice with metastasis. Notably, tumour-infiltrated areas were devoid of Ly6G^+^ cells while abundant Ly6G^+^ cells were observed in the tumour-free stroma (Supplementary Fig. 4g). As neutrophils were excluded from the infiltrated site, G-CSF released by 4T1.2 metastases is unlikely to trigger vessel remodelling via these haematopoietic cells.

Macrophages have also been implicated in sprouting angiogenesis and express *Csf3r*^37,38^. We assessed the requirement of these cells in vessel remodelling using an in vivo macrophage-ablation strategy, whereby mice inoculated with 4T1.2 cells were treated with anti-Csf1r Ab for 10 days (Fig. 6f). F4/80^+^ BM macrophages were reduced by 76% by the end of treatment (Fig. 6g and Supplementary Fig. 4h). 3D imaging and quantitative analysis found no difference in the abundance of vessel sprouting or bone metastatic burden between control and F4/80-depleted BM (Fig. 6h–j and Supplementary Fig. 4i, j). Thus, macrophages do not directly contribute to the transformation of BM vascular niches during lesion outgrowth.

To further exclude a role of hematopoietic lineages in mediating G-CSF-dependent vessel remodelling, we generated *Csf3r*^−*/*−^ BM chimeras by transplanting *Csf3r*^−*/*−^ BM into wild-type recipients (Fig. 6k). Exogenous G-CSF treatment significantly increased the number of vascular endothelial sprouts in *Csf3r*^−*/*−^ BM chimeras and vessel dilation, compared to their untreated counterparts (Fig. 6l–n). Notably, the fold increase in sprout density was similar to that measured in G-CSF-treated *Csf3r*^*+/+*^ mice (Figs. 4g and 6m). Together, these data provide evidence that G-CSF does not signal directly through hematopoietic intermediates to regulate vessel morphogenesis.

### Pharmacological interception of G-CSF signalling within the niche suppresses bone metastatic progression

**Fig. 7:**
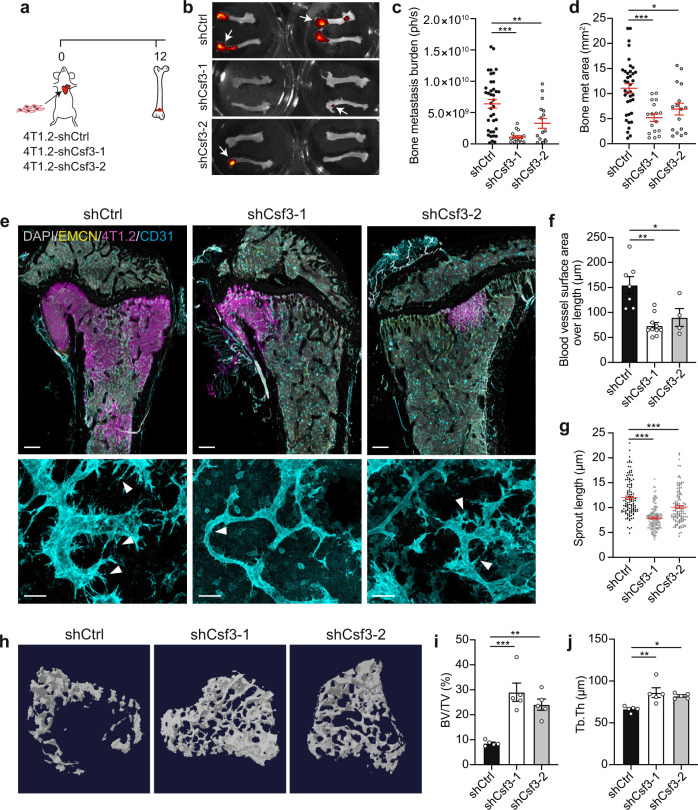
G-CSF deficiency suppresses remodelling of the BM vascular niche and bone metastasis. **a** Strategy to test the bone metastatic ability of G-CSF-deficient 4T1.2 cells. **b** Ex vivo fluorescence imaging of bones collected at experimental endpoint. Arrows point to metastasis in bone. **c**, **d** Quantification of photon flux (**c**) and bone metastasis area (**d**) from (**b**). Individual dots are from one bone. *n* = 11 (shCtrl) and 8 mice (shCsf3-1, shCsf3-2). In (**c**), **P = 0.0080, ***P < 0.0001. In (**d**), *P = 0.0126, ***P = 0.0003. *P* values, one-way ANOVA and Dunnett’s multiple comparisons test. **e** Representative 3D images (top) and enlarged micrographs (bottom) of femoral BM from mice injected with 4T1.2-shCtrl, 4T1.2-shCsf3-1 or 4T1.2-shCsf3-2 cells. Bones were stained for DAPI (grey), 4T1.2 cells (magenta), EMCN (yellow) and CD31 (cyan) (*n* = 5 mice per cell line). Arrowheads indicate endothelial sprouts. Scale bars: 300 µm (overviews), 10 µm (enlargements). **f**, **g** Quantification of surface area over length (**f**) and sprout length (**g**) of blood vessels from (**e**). Individual dots are from one vessel (**f**) or sprout (**g**). *n* = 3 mice per group. *P = 0.0116, **P = 0.0002, ***P < 0.0001 by one-way ANOVA and Dunnett’s multiple comparisons test. **h**–**j** Representative micro-computed tomography images (**h**) and morphometric analysis of tibial trabecula from metastasis-bearing mice. Data are presented as percentage of bone volume/tissue volume (BV/TV; %) (**i**) and trabecular thickness (Tb.Th; µm) (**j**). *n* = 5 mice per group. In (**i**), **P = 0.0016, ***P = 0.0002. In (**j**), *P = 0.0168, **P = 0.004. *P* values, one-way ANOVA and Dunnett’s multiple comparisons test. All data reflect mean ± s.e.m. Source data are provided as a Source Data file.

As tumour-derived G-CSF creates a metastasis-conducive BM microenvironment through vessel remodelling, we next assessed whether suppression of G-CSF activity could mitigate niche alteration and reduce bone metastasis in vivo. Two independent short hairpin RNA (shRNA) targeting *Csf3* were demonstrated to reduce G-CSF production in 4T1.2 cells without affecting tumorigenic function in vivo (Supplementary Fig. 5a–c). Intracardiac injection of *Csf3* knockdown (KD) cells dramatically reduced bone metastasis burden based on biofluorescence imaging (Fig. 7a–d). Moreover, 3D imaging of bone lesions from *Csf3* KD cancer cells revealed a substantial reduction in vascular sprouting frequency and length (Fig. 7e–g). Normalisation of the vascular niche also diminished metastasis-driven osteolysis and preserved metaphyseal trabecular bone structure (Fig. 7h–j).

**Fig. 8:**
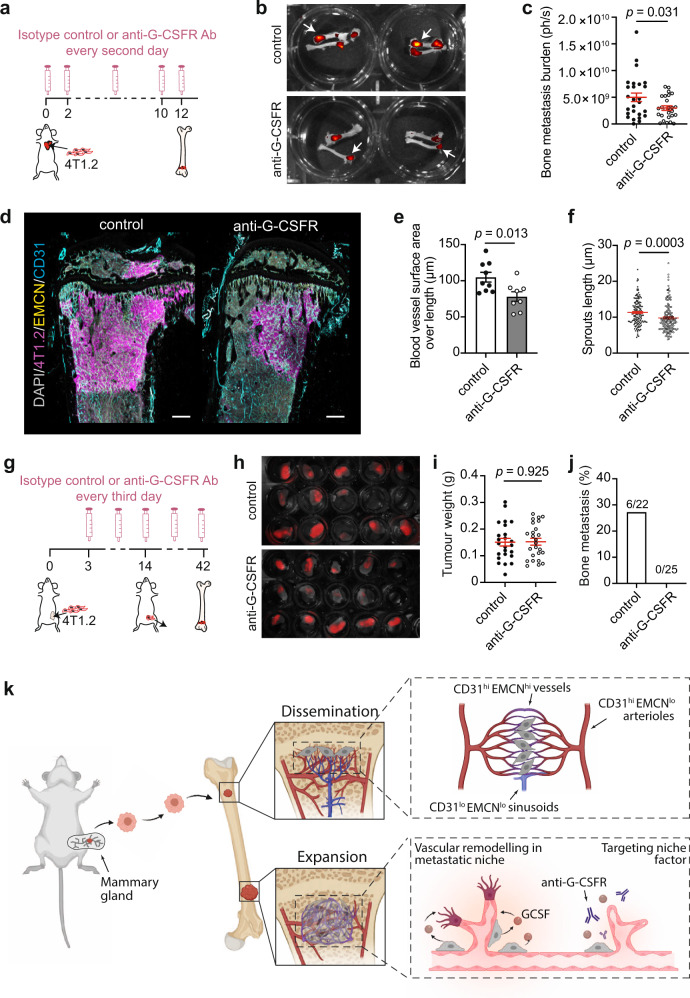
G-CSFR blockade protects against vessel remodelling and impedes experimental and spontaneous bone metastasis. **a** Strategy to evaluate the effect of anti-G-CSFR neutralising antibody (Ab) in the 4T1.2 experimental bone metastasis model. **b** Ex vivo fluorescence imaging of bones collected at experimental endpoint. Arrows point to metastasis in bone. **c** Quantification of photon flux from (**b**). Individual dots are from one bone (*n* = 9 mice per group). *P* value, two-tailed unpaired *t* tests. **d** 3D images of bone lesions in mice that received isotype control or anti-G-CSFR Ab. Bones were stained for DAPI (grey), 4T1.2 cells (magenta), EMCN (yellow) and CD31 (cyan) (*n* = 6 mice per group). Scale bars, 300 µm. **e**, **f** Quantification of surface area over length (**e**) and sprout length (**f**) of blood vessels from (**d**). Individual dots are from one vessel (**e**) or sprout (**f**). For (**e**), *n* = 4 (control) and 6 mice (anti-G-CSFR). For (**f**), *n* = 3 (control) and 4 mice (anti-G-CSFR). *P* values, two-tailed unpaired *t* tests. **g** Workflow to test the effect of anti-G-CSFR Ab in the 4T1.2 spontaneous bone metastasis model. **h**, **i** Ex vivo fluorescence imaging (**h**) and weight (**i**) of primary tumour at the time of surgical resection. *n* = 24 (control) and 25 mice (anti-G-CSFR). *P* value, two-tailed unpaired *t* tests. **j** Incidence of bone metastasis at experimental endpoint. **k** Schematic model of the vascular route exploited by mammary tumour cells for bone invasion and the role of G-CSF in creating a metastasis-favouring vascular niche. Image created using BioRender.com. All data reflect mean ± s.e.m. Source data are provided as a Source Data file.

We next explored whether pharmacological blockade of G-CSF signalling influenced the development of bone metastasis by treating BALB/c mice bearing advanced bone lesions with anti-G-CSFR Ab every third day for 1 week (Supplementary Fig. 5d). G-CSFR inhibition potently suppressed tumour-induced expansion and mobilisation of host neutrophils in peripheral blood and BM with variable impact on monocytes (Supplementary Fig. 5e–h). Accordingly, BM neutrophils exhibited reduced CXCR2 expression, consistent with a less activated phenotype (Supplementary Fig. 5i, j). Moreover, splenomegaly, an indicator of neutrophilia, was reversed in metastasis-burdened mice (Supplementary Fig. 5k). Confocal imaging and quantitative analysis of digitised vessels demonstrated that G-CSFR blockade attenuated vascular sprouting in bone macrometastases but did not rescue the narrowed lumens and reduction of vascular density at the site of macroscopic lesions (Supplementary Fig. 5l–p). The incidence and size of bone metastases were also comparable between treatment groups (Supplementary Fig. 5q, r). The ineffectiveness of G-CSFR blockade in suppressing overt bone lesions prompted us to speculate whether established metastases may become independent of niche signals for growth. We therefore modified the treatment regimen with the aim of targeting the initial steps of metastasis (Fig. 8a). Early anti-G-CSFR Ab administration at the time of intracardiac delivery of 4T1.2 cells resulted in substantially slower bone metastatic progression, as seen by 3D fluorescence imaging (Fig. 8b–d). Consistent with observations for *Csf3* KD mammary cancer cells, pharmacological inhibition of G-CSFR alleviated vessel remodelling in macroscopic bone lesions and offered modest protection against tumour-induced bone degradation (Fig. 8e, f and Supplementary Fig. 6a–d).

To test whether the bone metastasis-inhibiting effect of G-CSFR blockade could be replicated in a spontaneous model of metastasis, primary tumours were established by implanting 4T1.2 cells into the mammary fat pads of BALB/c mice. Three days later, mice were randomly grouped for treatment with isotype control or anti-G-CSFR Ab for 6 weeks (Fig. 8g). Consistent with a previous report^36^, anti-G-CSFR Ab treatment effectively reduced the incidence of multi-organ metastasis without affecting primary tumour growth (Fig. 8h–j and Supplementary Fig. 6e–g). The severity of lung and liver metastasis (determined by the number of tumour nodules), however, was unaffected by G-CSFR inhibition (Supplementary Fig. 6h, i). Remarkably, none of the anti-G-CSFR Ab-treated mice developed bone metastases (Fig. 8j), suggesting that G-CSF has a more defined role in promoting bone metastasis. Together these data show that early anti-G-CSFR administration in mice either intracardially or orthotopically injected with 4T1.2 cells effectively curtails vascular remodelling and metastatic outgrowth.

Finally, we assessed the clinical effectiveness of anti-G-CSFR Ab therapy in limiting distant metastases by comparison with docetaxel, a first-line chemotherapeutic agent for patients with metastatic breast cancer^39^, in the spontaneous metastasis model (Supplementary Fig. 6j). As expected, docetaxel alone markedly inhibited tumour growth at early time points but resistance to chemotherapy eventually emerged, resulting in widespread metastases (Supplementary Fig. 6k, m). As observed above, anti-G-CSFR monotherapy had limited efficacy in the early phase of tumorigenesis. However, subsequent disease progression was attenuated (potentially by suppressing tumour angiogenesis^40^), and achieved comparable survival to cohorts treated with docetaxel (Supplementary Fig. 6l). Furthermore, the neutralising Ab was more effective in suppressing distant metastasis than standard chemotherapy (Supplementary Fig. 6m). Notably, bone metastasis was potently inhibited by anti-G-CSFR Ab, with only one mouse showing a small cluster of DTCs at the femoral metaphysis (Supplementary Fig. 6n). The therapeutic response was not further enhanced by combining docetaxel with anti-G-CSFR Ab. In contrast, the beneficial effects of anti-G-CSFR antibody on bone and liver metastasis were not observed, possibly because chemotherapy treatment has been shown to damage the bone vascular network^41^ and in turn potentially compromised the anti-vascular remodelling effect of blocking Ab treatment (Supplementary Fig. 6k–m). Overall, the therapeutic inhibition of G-CSF-dependent vessel remodelling may represent a potential strategy to impede bone metastasis.

## Discussion

The success of metastatic colonisation requires complex interplay between tumour cells and the local microenvironment. In particular, the vascular endothelium has been implicated in the regulation of tumour cell quiescence and reactivation, and chemoprotection of indolent tumour cells in the BM^13,14^. However, relatively little is known about the cross-talk between metastatic solid tumour cells and the host BM microenvironment during lesion outgrowth. By applying large-scale quantitative 3D imaging to bone metastases, we show that mammary tumour cells home to a distinct vascular domain in the BM and initiate de novo formation of a pro-tumorigenic metastatic niche through remodelling of the local vasculature. Genetic and pharmacologic interception of tumour-derived G-CSF ameliorated the transformation of host niches and progression of breast cancer bone metastases (summarised in Fig. 8k).

Our data show that metastatic breast cancer cells specifically home to type H (or transitional) vessels, irrespective of species origin, the route of injection or integrity of the host immune system. Interestingly, phenotypically similar vessels defined by high expression of EMCN and CD31 (with unknown function) are present in liver, but are largely absent from other common sites of solid tumour metastasis including brain, lung and kidney^19^. This suggests that type H vessels constitute a distinct vascular microenvironment that supports solid tumour engraftment in the BM. Anatomically, type H vessels connect arteries with sinusoids and represent a site where blood-flow drops dramatically and with privileged access to oxygen and nutrients^42,43^. Furthermore, type H vasculature stimulates bone-forming osteoprogenitors through the secretion of Noggin^44^ and produces stem cell factor^45^ (a cytokine crucial for hematopoietic stem cells (HSC) longevity^46^). In line with the notion that the stem cell niche can support preferential expansion of tumour cells, different studies have documented the trafficking of leukaemia^25^, prostate^47^ and breast tumour cells^48^ to HSC niches in the BM and the role of these microenvironments in driving the growth, survival and long-term protection of these cells. It is therefore conceivable that the anatomical features and the abundant resource of growth factors and cytokines provided by type H vessels could represent a fertile ground for the seeding of metastatic cells.

Apart from serving as molecular parasites of the host niche, an emerging concept in the field is that tumour cells are capable of transforming the surrounding stroma in a lineage-specific manner to create a favourable microenvironment that supports their proliferation and survival, namely the metastatic niche. For instance, T-cell acute lymphoblastic leukaemia (T-ALL) infiltration was shown to rapidly deplete osteogenic cells in the BM^49^, whereas acute myeloid leukaemia (AML) engraftment caused EC sprouting and collapse of the endosteal vessels at late stage disease^23^. Preserving endosteal vessels of the leukaemia BM improved the efficacy of chemotherapy treatment^23^, raising the possibility of targeting the microenvironment to restrict tumour cell growth. Here we extend findings described for haematological malignancies to solid cancer metastasis, in which disseminated mammary tumour cells were shown to transform the BM vascular niche for expansion. The phenotypic characteristics of vessels remodelled by mammary metastatic lesions, namely endothelial disorganisation, compression, and sprouting were remarkably similar to those identified in leukaemia BM, implying a shared stromal response to both liquid and solid cancer cell occupancy. Another notable molecular signature of the bone metastasis-remodelled vessels is their high expression of EMCN and CD31. Despite sharing similar biomarkers with metaphyseal type H vessels, they lack coverage of pericytes or osteoprogenitors, suggesting that the remodelled vascular network comprises a mix of type H and sinusoidal vessels. While the exact function of these remodelled vessels is unclear, damage to the local vascular network presumably affects local blood flow, hinders immune cell infiltration, and reduces the supply of nutrients (as well as anti-cancer agents), thus promoting metastatic outgrowth^50^.

ECs in tumour-infiltrated BM regions appear to be enriched for angiogenic and inflammation response gene signatures. Transcriptomic profiling of 4T1.2 tumour cells revealed the overexpression of extracellular factors with known roles in angiomodulation, including *Fgf1*^31^*, Pdgfb*^32^ and *Dll4*^33^. The contact-dependent Dll4-Notch interaction between endothelial cells has been demonstrated to specify endothelial tip cell identity and coordinate sprouting angiogenesis^33,51^. In non-skeletal organs and malignant tumours, Dll4 acts as a potent inhibitor of vascular growth and sprouting^52^. In contrast, endothelial Dll4 promotes angiogenesis in long bones and enhances type H vessel growth^44^. It is unclear whether tumour cell-derived Dll4 exploits this organ-specific angiogenic mechanism to facilitate BM vessel remodelling. However, other pro-inflammatory molecules such as CXCL2 and TNF^23^, which have been implicated in the AML-induced vascular remodelling process, are not expressed in 4T1.2 cells. In the context of breast cancer bone metastases, we provide evidence that G-CSF is a key tumoral factor that triggers vessel remodelling in the host BM. Only the G-CSF-secreting line 4T1.2 and not the non-producing isogenic line 67NR stimulated vascular sprouting in BM metastatic lesions. Moreover, the administration of G-CSF to mimic tumour infiltration was sufficient to elicit widespread endothelial sprouting throughout the BM in tumour-naïve mice. Most importantly, the overexpression of *Csf3* in EMT6.5 mammary tumour cells or ablation of *Csf3* in 4T1.2 cells was found to promote or diminish BM vessel remodelling, respectively. The ability of G-CSF to modulate BM endothelium in vivo is reminiscent of prior work indicating that this cytokine can stimulate tumour angiogenesis by a mechanism that is not fully understood^40^. Bone marrow chimera experiments together with the genetic- and antibody-mediated strategies that we employed to ablate leucocyte subsets all demonstrated that hematopoietic cells are not necessary for G-CSF-initiated BM vessel remodelling. Although the possibility that G-CSF may impact on a mesenchymal constituent to reprogram vascular niche cannot be excluded, the observations that vessel remodelling is specific to tumour-infiltrated areas and that endothelial cells express G-CSFR^37,53^ suggest that tumour-derived G-CSF directly activates BM ECs to induce vascular remodelling.

Increasing evidence indicates that G-CSF plays a pro-metastatic role in breast cancer pulmonary metastasis. This has been predominantly attributed to the ability of G-CSF to: (i) recruit myeloid-derived suppressor cells and neutrophils, which suppress T-cell and NK-cell cytotoxic activity^34,54,55^; (ii) mobilise granulocytes to initiate pre-metastatic niche formation^35^; and (iii) release neutrophil extracellular traps that enhance tumour cell mobility^56^. Our study has uncovered a role for G-CSF biology in bone metastasis, in which the cytokine appears to remodel the BM vasculature in a hematopoietic cell-independent manner to create a pro-metastatic microenvironment. Targeting the niche remodelling factor via blockade of G-CSF signalling alleviated pathological blood vessel remodelling and significantly impeded bone metastasis progression in both experimental and spontaneous metastasis models. Strikingly, G-CSFR neutralising Ab totally abrogated spontaneous breast cancer bone metastasis while lung and liver metastases were also reduced, albeit to a lesser degree, suggesting that G-CSF has a prominent role in facilitating bone-tropism of breast cancer cells. Consistent with the angio-stimulatory role for G-CSF unveiled here, G-CSF was found to enhance melanoma and mammary tumour growth in bone in an osteoclast-dependent manner^57^. G-CSF-expanded neutrophils have been implicated in metastatic colonisation of lung through formation of a pre-metastatic microenvironment^35^. However, we found that Ly6G^+^ neutrophils did not localise to the tumour-infiltrated BM area (in fact, they appeared to be completely excluded) and antibody-based neutrophil depletion did not hinder bone lesion expansion. Consistent with these findings, G-CSF but not AMD3100, an alternative neutrophil mobilising agent, was shown to stimulate tumour growth in bone^57^, suggesting that neutrophils do not play a significant role in bone metastasis.

Recombinant G-CSF has been approved for treating chemotherapy-induced neutropenia in cancer patients with little evidence linked to increased cancer metastasis. A plausible explanation is provided by Li et al.^55^ who demonstrated that exogenous G-CSF displayed pro-metastatic and anti-metastatic effects in the presence or absence of NK cells, respectively. As such, patients with impaired NK cell function induced by cytotoxic therapy could experience a net anti-metastatic outcome upon G-CSF administration. In addition, the supraphysiological level of G-CSF secreted by tumour cells likely exceeds the clinically regulated dose that patients receive. Hence, our results suggest that careful evaluation of tumoral G-CSF levels may identify individuals who might benefit from G-CSFR blockade.

This study highlights the dynamic nature of the BM microenvironment and the ability of infiltrating solid tumour cells to rapidly remodel the bone marrow niche to create a self-sustaining microenvironment. G-CSFR blockade had minimal impact on advanced bone lesions, in contrast to its effect on early lesions, indicating that G-CSF-mediated vascular remodelling is critical for the initial colonisation stage by tumour cells. It is therefore important to consider the co-evolution of tumour cells and their microenvironment during metastatic progression in order to define the critical therapeutic window when designing niche-targeting approaches. While G-CSF seems to be a key driver of BM vessel remodelling and metastasis, non-G-CSF producing breast tumour cells (both 67NR and EMT6.5) could also give rise to bone lesions (with delayed kinetics), suggesting that alternative cell-intrinsic or niche-transforming mechanisms support their propagation in bone. As exemplified by the diverse niche-remodelling factors across multiple leukaemias^22–26^, we speculate that most microenvironment interactions are cell-type specific. Future work will be required to determine whether reprogramming of the BM vasculature by tumour-derived G-CSF applies to other solid malignancies. Targeting pathological features associated with niche deregulation represents a potential therapeutic avenue for limiting bone metastasis that is likely to be applicable to diverse tumour types.

## Methods

### Cell culture and reagents

The 67NR, 66cl4, 4T1, 4T1.2 murine mammary tumour cell lines, and their fluorescent reporter derivatives were maintained in α Minimal Essential Medium (αMEM) and 5% FCS. The EMT6.5-mCherry murine mammary tumour cell line was maintained in Dulbecco’s modified Eagle medium (DMEM) and 10% FCS. The human cell line MDA-MB-231 was obtained commercially (ATCC) and cultured in RPMI-1640 medium supplemented with 1% HEPES and 10% FCS. To generate a stable fluorescent reporter derivative, the 4T1.2 cell line was stably transfected with pTol2.RFP expression plasmid (gift from C. Marcelle, Monash University) and transposase plasmid, using Lipofectamine LTX with PLUS reagent. MDA-MB-231 cells were transduced with pFU-Luc2-eGFP lentiviral particles (kindly provided by D. Merino, WEHI) cells. The MDA-MB-231-GFP cell line was generated by C. Dawson. To overexpress *Csf3*, EMT6.5-mCherry cells were infected with retroviral particles containing the *Csf3* expression construct and selected with puromycin. To knockdown *Csf3*, 4T1.2 cells were infected with lentiviral particles containing *Csf3*-targeting shRNA expression constructs (Sigma) and selected with puromycin. Conditioned cell media were harvested for ELISA assay for G-CSF as per the manufacturer’s instruction (R&D Systems). All cell lines were shown to be mycoplasma-free.

### Mouse experiments

Wild-type BALB/c, *Csf3r*^−*/*−^ and NOD-SCID mice were bred and maintained by the animal facility of the Walter and Eliza Hall Institute (WEHI). *Col2.3-CFP* mice were provided by D. Rowe (University of Connecticut). *Flk1-GFP* mice have been described^51^. All animal work was performed in accordance with regulatory standards and were approved by the WEHI Animal Ethics Committee and the Austin Health Animal Ethics Committee. Orthotopic transplantation and intracardiac injections were performed as previously described^10^. Briefly, 10^5^ and 10^6^ viable cells were injected into the inguinal mammary gland and left ventricle, respectively. In the case of orthotopic transplantation, primary tumours were resected when they reached 200 mm^3^ (at around 2 weeks post transplantation). Tumour volume was calculated by measuring the length and width of the tumour with digital vernier calipers and using the formula: (width)^2^(length)/2. Neutrophil depletion by anti-Ly6G antibody was performed by intraperitoneal injection of 100 µg 1A8 or rat IgG2a isotype control (WEHI antibody facility) at the indicated intervals. Macrophage depletion by anti-Csf1r antibody was performed by intraperitoneal injection of 300 µg AFS98 or rat IgG2a isotype control (WEHI antibody facility) at the indicated intervals. To neutralise G-CSFR activity, mice were administrated intraperitoneally with 50 µg (mammary fat pad experiment) or 100 µg (intracardiac experiment) mouse anti-G-CSFR (clone Ch5E2-VR81, CSL limited) or IgG1k isotype control antibody (clone BM4, CSL limited) at the indicated intervals. For the anti-G-CSFR Ab and docetaxel combination treatment, 4T1.2 tumour-bearing mice were randomly grouped and subsequently treated with 50 µg mouse anti-G-CSFR or mouse IgG1K isotype control antibodies every third day, from days 7 to 25 after orthotopic transplantation. Docetaxel (15 mg/kg) or vehicle (5% ethanol, 5% Tween-80 in PBS) were delivered intraperitoneally at days 10, 19 and 28 post transplantation. In all animal studies, mice were monitored daily for respiratory distress or signs of paralysis. At the experimental endpoint, mice were euthanised and organs and primary tumours (if not resected) were collected for histological analysis. All mice used in this project were housed in plastic cages between 20–22 °C and 40–70% humidity under a 14-h light/10-h dark cycle.

### 3D confocal imaging of bone marrow

We adapted previously published protocols on BM imaging^16,17^. In brief, freshly dissected bones were fixed immediately in ice-cold 4% paraformaldehyde/PBS solution for 16 h. Bones were decalcified with 0.5 M EDTA in H_2_O with constant rolling at 4 °C for 24 h. Following decalcification, bones were incubated in ice-cold 20% sucrose-2% polyvinylpyrrolidone PBS solution for 24 h, and embedded in PBS containing 20% sucrose, 8% gelatin and 2% polyvinylpyrrolidone for storage at −80 °C. Thick bone cryosections (100−300 µm) were generated using feather C35 microtome blades on a Thermo Scientific HM525 cryostat. Freshly prepared cryosections were either directly immersed into PBS or mounted on gelatin-coated glass slides for long-term storage.

For immunostaining, cryosections were rehydrated with PBS at 37 °C to remove the embedding medium, permeabilised with 0.3% Triton-X/PBS for 20 min, blocked in PBS containing 20% DMSO and 10% donkey serum overnight at room temperature. Primary antibodies were incubated overnight at room temperature. Primary antibodies included: Endomucin (rat, clone V.7C7, Santa Cruz, 1/100 dilution), CD31 (goat polyclonal, R&D Systems, 1/50 dilution), RFP (rabbit polyclonal, Rockland, 1/1000 dilution), GFP (chicken polyclonal, Abcam, 1/400 dilution), Collagen Type I (rabbit polyclonal, Merck Millipore, 1/100 dilution), Keratin-8/18 (rat, clone TROMA-I, DSHB, 1/250 dilution), Osterix (rabbit polyclonal, Abcam, 1/1200 dilution), Cleaved caspase-3 (rabbit, clone 5A1E, Cell Signaling, 1/200 dilution), Ki67 (rabbit, clone D3B5, Cell Signaling, 1/100 dilution), PDGFRβ (rabbit, clone 28E1, Cell Signaling, 1/100 dilution). Conjugated antibodies included: SMA Alexa Fluor 488 (mouse, clone 1A4, R&D Systems, 1/50 dilution), Ly6G Brilliant Violet 510 (rat, clone 1A8, Biolegend, 1/100 dilution). Following primary antibody staining, sections were washed and then incubated with secondary antibodies in blocking buffer (1/400 dilution) overnight at room temperature. Secondary antibodies: anti-rat Alexa Fluor 488 (Cat. No. A21208, Invitrogen), anti-rat Alexa Fluor 594 (Cat. No. A21209, Invitrogen), anti-rat Alexa Fluor 647 (Cat. No. A21247, Invitrogen), anti-rabbit Alexa Fluor 488 (Cat. No. A21206, Invitrogen), anti-rabbit Alexa Fluor 555 (Cat. No. A31572, Invitrogen), anti-rabbit Alexa Fluor 647 (Cat. No. A32795, Invitrogen), anti-goat Alexa Fluor 488 (Cat. No. A11055, Invitrogen), anti-goat Alexa Fluor 647 (Cat. No. A21447, Invitrogen), anti-chicken Alexa Fluor 488 (Cat. No. A11039, Invitrogen) and DAPI (Sigma, 10 µg/ml) for nuclear staining. Sections were washed and then cleared in a graded series of 2,2ʹ-thiodiethanol in PBS (TDE, Sigma-Aldrich) over a period of 2 days and mounted in 100% TDE and sealed with coverslips.

Bone marrow imaging of cleared tissue (Figs. 4e, f, 6l and Supplementary Fig. 3b, d) was performed according to the previously published method by Gomariz et al.^21^. Bones were isolated and fixed in 2% paraformaldehyde/PBS overnight at 4 °C. Bones were dehydrated in 30% sucrose in PBS for 72 h at 4 °C, washed, then frozen in optimal cutting temperature (OCT, Tissue-Tek) solution and snap frozen in an isopropanol dry ice slurry. Bones were sectioned until the BM cavity was fully exposed along the longitudinal axis and then reversed and re-cut till the opposite face of BM was evenly exposed. Once BM slices were generated, OCT medium was removed by washing bone slices in PBS. For immunostaining, BM slices were incubated in blocking solution (0.2% Triton X-100, 1% bovine serum albumin (BSA) and 10% donkey serum in PBS) overnight at 4 °C. Primary antibody incubations were performed for 3 days at 4 °C followed by overnight washing in PBS. Secondary antibody staining was performed for 3 days at 4 °C in 0.2% Triton X-100 and 10% donkey serum in PBS, then washed overnight. Samples were washed in RapiClear 1.52 (Sunjin Lab) for 1–3 days and checked visually for clearing every day. All incubation and wash steps were performed with gentle rotation. Once cleared, samples were mounted on a standard microscopy slide, surrounded by a wall of vacuum grease, covered in RapiClear 1.52 and sealed with a coverslip for imaging. The clearing protocol typically increased the imaging depth to 300 µm from the tissue surface without significant loss of fluorescence intensity.

### Image acquisition and quantitative analysis

High-resolution immunofluorescence images were captured using laser scanning confocal microscopes (Zeiss LSM 780 and Zeiss LSM 880 Fast Airyscan) and Zeiss LSM 880 NLO multiphoton microscope. Tile scans of Z-stacks across the entire bone were imaged using spectral unmixing mode at 1024 × 1024 pixels with an objective Plan-Apochromat ×10/0.45 M27 or at 512 × 512 pixels with an objective W Plan-Apochromat ×20/1.0 DIC (UV) VIS-IR M27 75 mm. To obtain cellular resolution at regions of interest, an objective Plan-Apochromat ×40/1.3 DIC UV-IR M27 oil objective was used. A 32-channel GaAsP array and NDD detectors were deployed for simultaneous confocal and multiphoton imaging. Z-stacks of images were stitched using ZEN black v.2.3 (Zeiss). This was followed by visualisation and analysis in Imaris v.8.3 or v.9.3 (Bitplane) and FIJI/Image J v.2.0.0 (NIH).

For the determination of DTC localisation, DTCs were first identified automatically using the Imaris spots function and manually curated by scanning through optical sections. Trabecular and cortical bone surfaces were annotated based on Col1a1 immunolabelling or second harmonic signal and tissue morphology using the Imaris surface function. We primarily used CD31 staining to label the vasculature within the BM. To subdivide vessel subtypes, we incorporated the EMCN channel and defined arterial vessels as CD31^hi^EMCN^–^, type H vessels as CD31^hi^EMCN^hi^, and sinusoids as CD31^lo^EMCN^lo^ as previously reported^19^. Since we imaged >100 µm of the marrow cavity, we were able to trace each arteriole and its subsequent branching into type H vessels and sinusoids. The preservation of vessel continuity allowed us to unambiguously distinguish vessels in a way that is not achievable in thin sections. Therefore, based on vessel orientation and markers expression, we created distinct digital surfaces corresponding to each blood vessel subtype using the Imaris signal colocalisation, channel arithmetic and surface function. 3D distances between individual DTCs and digital vessels or bone were then computed using the Imaris distance transformation XTension program.

For random spots generation and measurements, 3D images were imported into FIJI and a threshold was set to exclude blood vessel lumens and bone surfaces where DTCs were not found, and random spots were not generated. Spots matching the size of DTCs (10 µm) were placed at random *x,y,z* coordinates using a previously published FIJI macro^49,58^. To avoid over- or underestimation of spatial interactions between simulated spots and BM landmarks, we only generated random spots on image stacks of DTCs that contain all relevant blood vessel subtypes, namely arterioles, sinusoids, type H vessels and bone. We empirically tested the randomness of the distribution and the minimal number of random spots required to create a reliable simulated dataset. The simulated spots were then imported back to Imaris, converted to spheres, and their distance to landmarks in the BM were computed using the Imaris distance transformation module.

For the measurement of tumour size in primary and secondary lesion sites, FIJI was used by thresholding RFP signal (derived from anti-RFP antibody against cancer cells) of maximum intensity projected images. Intralesional vessel density was determined by masking CD31 signal using RFP signal in Imaris, and then measuring the volume occupied by CD31 vessels and dividing this by the volume of tumour.

For quantification of blood vessel morphology, the endothelial lumen area was measured in Imaris by manually outlining the lumen in cross-sectional plane of CD31^+^ blood vessels. The blood vessel surface area over length was determined by creating surfaces using the CD31 channel, measuring the area using Imaris surface function, and then manually calculating the vessel length using Imaris measurement points function and dividing the two values. The length of vascular sprouts was determined by manually annotating thin, CD31-immunoreactive projections extended from the endothelial basement membrane using the Imaris measurement points function.

### Ex vivo fluorescence imaging and micro-computed tomography (µCT) analysis

Freshly harvested organs were fluorescently imaged using the IVIS Lumina S5 platform (PerkinElmer). Images that were used for direct comparisons were acquired using the same field of view, exposure time, pixel binning and f-stop settings and analysed by the Living image software (PerkinElmer). For µCT analysis, 4% paraformaldehyde/PBS solution fixed bones were scanned with the Bruker Skyscan 1276 system at a resolution of 17.56 µm/pixel. 3D structural analyses were completed using the CTvol and CTAn software (Bruker).

### Flow cytometry

To analyse circulating immunocytes, cells of retro-orbital blood were treated with red blood cell removal buffer for 1 min at room temperature, resuspended in PBS with 2% FCS (FACS buffer). Immunostaining was performed at 4 °C as follows: the cells were blocked with 0.1 mg/ml anti-mouse IgG2a (rat, clone GL117, in-house, 1/80 dilution) and anti-CD16/32 (rat, clone 2.4G2, in-house, 1/40 dilution), and then incubated with antibodies against CD45 APC (rat, clone 30-F11, Biolegend, 1/100 dilution), CD11b Brilliant Violet 785 (rat, clone M1/7, Biolegend, 1/4000 dilution), Gr-1 FITC (rat, clone RB6-8C5, Biolegend, 1/400 dilution), Ly6G Brilliant Violet 510 (rat, clone 1A8, Biolegend, 1/50 dilution) and Ly6C PE-Cy7 (rat, clone HK1.4, Biolegend, 1/600 dilution) for 30 min. After staining and washing, samples were resuspended in FACS buffer containing 7-AAD (0.2 µg/ml, Sigma) or propidium iodide (PI, 0.5 µg/ml, Sigma) to exclude dead cells during flow cytometry. For hematopoietic cell analysis of BM, freshly harvested femurs were crushed in ice-cold FACS buffer and filtered through a 40 µm strainer. The resulting cell suspension underwent the same staining procedure as described above.

Flow cytometry analysis of bone marrow (BM) endothelial cells was performed as previously described^23^. In brief, tibias and femurs were cleaned thoroughly to remove adherent muscles. Bones were crushed in ice-cold FACS buffer with pre-chilled mortar and pestle. Whole BM (including bone pieces) were digested with collagenase I (Cat. No. LS004196; Worthington) at 37 °C for 20 min with agitation at 120 rpm, and the suspension were filtered through a 100 µm strainer. Following a 1-min treatment with red blood cell removal buffer at room temperature, the cells were filtered through a 40 µm strainer to obtain a single-cell suspension. CD45 APC (rat, clone 30-F11, Biolegend, 1/100 dilution) and Ter-119 APC (rat, clone TER-119, Biolegend, 1/100 dilution) antibodies were used to gate out hematopoietic cells; CD31 PE-Cy7 (rat, clone 390, Biolegend, 1/100 dilution) and EMCN Alexa Fluor 488 (rat, clone V.7C7, Santa Cruz, 1/50 dilution) antibodies were used to isolate endothelial cells. Antibody staining of cell suspension was performed at 4 °C for 45 min. Following washing, cells were resuspended with FACS buffer containing DAPI (0.1 µg/ml, Sigma) to mark dead cells. A similar procedure without the digestion step was used to analyse BM immune cells. Antibodies recognising CD45 Brilliant Violet 605 (rat, clone 30-F11, Biolegend, 1/200 dilution), CXCR2 PE (rat, clone SA044G4, Biolegend, 1/200 dilution), F4/80 APC/Cy7 (rat, clone BM8, Biolegend, 1/200 dilution) and CD115 APC (rat, clone AFS98, Biolegend, 1/300 dilution) were used. In some experiments, beads of known concentration were added to determine absolute cell counts. Samples were analysed on a LSR Fortessa or sort-purified using a FACS Aria with FACSDiva v.8.0 (BD Biosciences) and data were analysed with FlowJo v.9 or v.10 (Tree Star).

### G-CSF mouse injections and *Csfr3*^−*/*−^ BM chimeras

*Flk1-GFP* mice were injected subcutaneously twice daily for 4 days with 250 µg/kg Filgrastim diluted in PBS. Bones were harvested on day 4 after second injection. To generate *Csf3r*^−*/*−^ chimeric mice, *Flk1-GFP* recipient mice were irradiated with two doses of 550 Rads (5.5 Gy) >3 h apart. Irradiated mice received 1 × 10^7^ bone marrow cells isolated from age- and sex-matched *Csfr3*^−*/*−^ or *Csfr3*^*+/+*^ littermates. Ly5.1+ recipients were irradiated and transplanted to determine the reconstitution efficiency.

### RNA sequencing of bone marrow ECs

Bones from tumour-burdened mice were microdissected to separate tumour-infiltrated marrow (met) from adjacent tumour-free marrow (non-met) based on RFP fluorescence signal; CD31^hi^EMCN^hi^ ECs were then sorted from the BM of 8–10-week-old BALB/c mice (3–4 pools of 1−4 mice) as described above. The same ECs subset was also sorted from three pools of age-matched, non-tumour-burdened BALB/c mice as controls. Total RNA from 700 to 5500 cells was used to generate libraries for whole-transcriptome analysis following the Clontech v4 low input RNA protocol. Libraries were sequenced on an Illumina NextSeq 500. Between 7 and 38 million 75 bp paired-end reads were generated for each sample. Reads were aligned to the mouse genome mm10 using Rsubread version 1.34.4 ^59^. One sample with a low mapping percentage (<20% read-pairs successfully aligned) was removed, leaving three met samples, six non-met and two control samples. The number of reads overlapping each Entrez gene were counted using featureCounts and Rsubread’s built-in NCBI annotation^60^. Gene information was downloaded from the NCBI (2 February 2019). Downstream analysis used the limma^61^ and edgeR^62^. Gene filtering was performed using edgeR’s filterByExpr function with default settings. Ribosomal genes, predicted genes, unassembled contigs and obsolete Entrez Gene IDs were removed from further analyses. Differential expression analysis was performed using the edgeR’s voomLmFit function^63^ with empirical sample weights^64^. Genes were considered to be differentially expressed if they achieved a false discovery rate (FDR) below 5%. Gene ontology analysis was performed using limma’s goana function. Log2-RPKM values for each gene across samples were calculated using edgeR’s rpkm function with a prior count of 2. Heat maps were generated using the heatmap.2 function of the gplots package. Log2-RPKM values were standardised to have mean 0 and standard deviation 1 for each gene before producing the heatmaps, after which genes and samples were clustered by the Ward’s minimum variance method.

### RNA sequencing of tumour cell lines

Total RNA was extracted from mammary tumour cell lines 4T1, 4T1.2, 67NR and 66cl4 for whole-transcriptome analysis following Illumina’s TruSeq RNA v2 sample preparation protocol (100 ng RNA as input), with two biological replicates for each. Libraries were sequenced and analysed similarly to the BmEC RNA-seq except that differential expression between the cell lines was assessed using edgeR’s quasi-likelihood pipeline^65^.

### Statistics and reproducibility

No statistical method was applied to predetermine sample size. No blinding was used. Data were organised using Excel v.16.30 (Microsoft) and statistical analyses were conducted using Prism v.9.0 (GraphPad). Error bars in all panels represent mean ± standard error of the mean (s.e.m.).

### Reporting summary

Further information on research design is available in the Nature Research Reporting Summary linked to this article.

## Supplementary information

Supplementary Information

Description of Additional Supplementary Files

Supplementary Movie 1

Supplementary Movie 2

Supplementary Movie 3

Supplementary Movie 4

Supplementary Movie 5

Reporting Summary

## Data Availability

The RNA-seq data that support the findings of this study have been deposited in the GEO under the accession code GSE160102. All raw images are available from the authors upon request. Source data are provided with this paper.

## Source data

Source Data

## Code availability

The code for random spot generation can be found in Zenodo [10.5281/zenodo.5527727].
