# Supplementary material for: Mammary tumour cells remodel the bone marrow vascular microenvironment to support metastasis
Source: Nat Commun. 2021 Nov 26;12:6920. doi: 10.1038/s41467-021-26556-6 (PMC8626461; doi:10.1038/s41467-021-26556-6)
Supplement: Supplementary file 1 — Supplementary Information [file 41467_2021_26556_MOESM1_ESM.pdf]

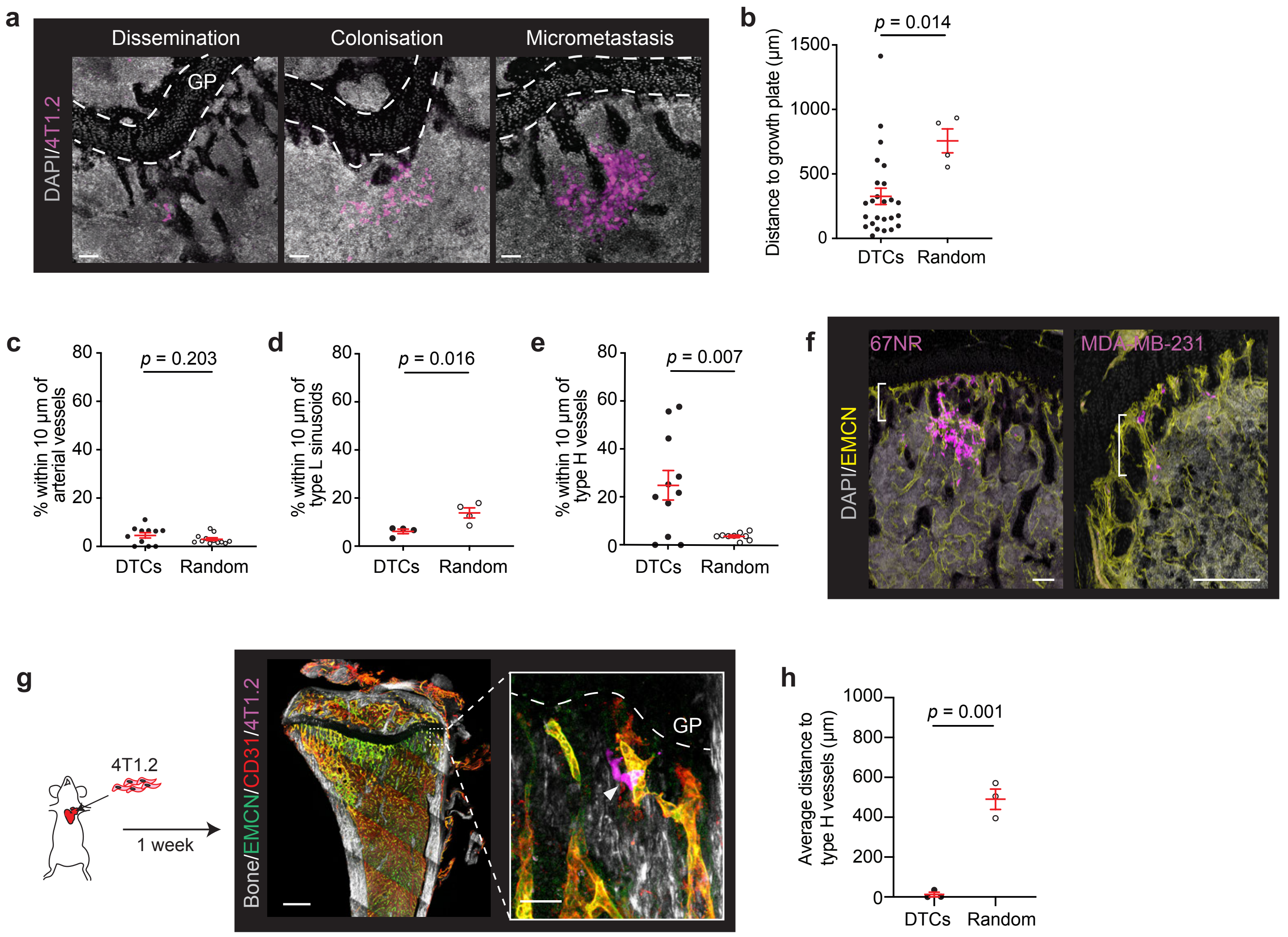

Supplementary Figure 1

Supplementary Fig. 1: In situ identification of the bone metastatic niche for mammary cancer. a, Confocal images of femoral and tibial bone marrow stained for DAPI (grey) and 4T1.2 disseminated tumour cells (DTCs; magenta) (*n*=3 mice per stage). Dotted lines demarcate growth plate (GP). Scale bars: 50 μm. **b**, The average distance to the growth plate for DTCs and simulated random spots from **a**. Individual dots correspond to the average values of DTCs or random spots from one bone. A total of 547 DTCs in 25 bone lesions were analysed in 11 mice. *P* value, two-tailed unpaired *t*-test. **c-e**, Percentages of 4T1.2 DTCs and random spots within 10 μm of an arterial vessel (**c**), a type L sinusoid (**d**), or a type H vessel (**e**). A total of 1,222 (**c**), 299 (**d**) and 1,027 (**e**) DTCs were analysed in 11 (**c**), 4 (**d**) and 11 (**e**) mice, respectively. *P* values, two-tailed unpaired *t*-tests. **f**, Representative 3D images of BM from mice that received intracardiac injections of 67NR (left) or MDA-MB-231 (right) cells and collected at 10 days and 15 days post-injection, respectively. Bones were stained for DAPI (grey), 67NR or MDA-MB-231 (magenta) and EMCN (yellow). Brackets mark metaphyseal type H endothelium (*n*=2 mice per cell line). Scale bars: 100 μm. **g**, Schematic diagram showing intracardiac injection experiment. Multiphoton 3D images of BM from an inoculated mouse, immunostained for 4T1.2 DTCs (magenta), EMCN (green) and CD31 (red). Bone collagen was defined by second harmonic signal (grey) (*n*=3 mice). Dotted line marks the growth plate (GP) boundary. Arrowhead points to DTCs. Scale bars: 500 μm (overview), 50 μm (enlargement). **h**, Distance to the nearest type H vessel of DTCs and simulated random spots from **g**. Individual dots are from metastases from different mice. A total of 36 DTCs were analysed in 3 mice. *P* value, two-tailed unpaired *t*-tests. All data reflect mean ± s.e.m. Source data are provided as a Source Data file.

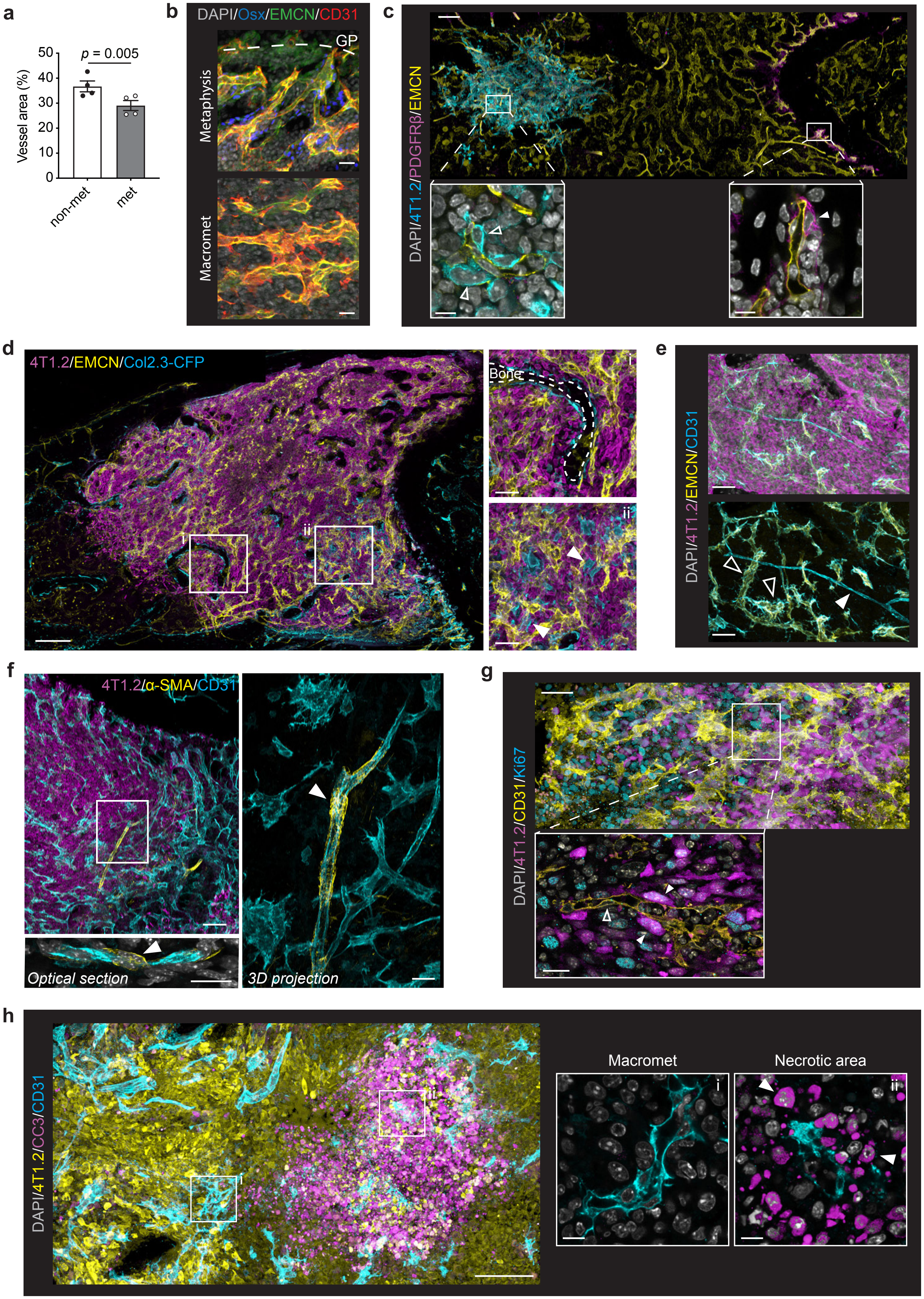

Supplementary Figure 2

Supplementary Fig. 2: Characteristics of remodelled vasculature in the BM metastatic niche. **a**, Percentages of BM covered by $CD31^+$ blood vessels in tumour-free (non-met) and adjacent tumour-infiltrated (met) areas (*n*=4 mice). *P* value, two-tailed paired *t*-test. **b**, 3D images of tumour-free metaphysis and macroscopic lesion of the same bone. Bones were stained for DAPI (grey), Osterix (blue), EMCN (green) and CD31 (red) (*n*=3 mice). Dotted line marks the growth plate (GP) boundary. Scale bars, 20 μm. **c**, Optical sections from 3D image of a 4T1.2 bone metastasis (cyan) stained for DAPI (grey), $PDGFR\beta^+$ (magenta) and EMCN (yellow) (*n*=3 mice). Filled arrowhead marks $PDGFR\beta^+$ pericytes covering metaphyseal $EMCN^{hi}$ endothelium. Open arrowheads mark tumour cells. Scale bars: 100 μm (overview), 10 μm (enlargements). **d**, 3D images of a 4T1.2 macrometastasis (magenta) in the marrow of a *Col2.3-CFP* (cyan) reporter mouse stained for EMCN (yellow) (*n*=3 mice). Enlargements of boxed regions are displayed on the right. Dotted line outlines bone surface. Arrowheads indicate detached CFP+ cells. Scale bars, 200 μm (overview) and 50 μm (enlargements). **e**, 3D images of a 4T1.2 macrometastasis (magenta) stained for DAPI (grey), EMCN (yellow) and CD31 (cyan) (*n*=10 mice). Filled arrowhead indicates $CD31^+EMCN^-$ arterial vessels and open arrowheads denote $CD31^{hi}EMCN^{hi}$ vessels with endothelial sprouts. Scale bars: 50 μm. **f**, Optical sections from 3D images of a 4T1.2 macrometastasis (magenta) immunostained for α-SMA (yellow) and CD31 (cyan) (*n*=3 mice). Arrowheads indicate α-SMA^+ cells covering arterial vessels. Scale bars: 100 μm (overview) and 20 μm (enlargements). **g**, Optical section from 3D image of a 4T1.2 macrometastasis (magenta) immunostained for CD31 (yellow) and Ki67 (cyan) (*n*=3 mice). Filled arrowheads mark proliferative 4T1.2 cells. Open arrowhead depicts $Ki67^–$ endothelial cells. Scale bars: 50 μm (overview), 20 μm (enlargement). **h**, Optical sections from 3D image of a 4T1.2 macrometastasis (yellow) immunostained for cleaved caspase 3 (CC3; magenta) and CD31 (cyan) (*n*=3 mice). A necrotic area of the lesion is shown as a positive control. Arrowheads indicate $CC3^+$ cells. Scale bars: 100 μm (overview), 10 μm (enlargements). All data reflect mean ± s.e.m. Source data are provided as a Source Data file.

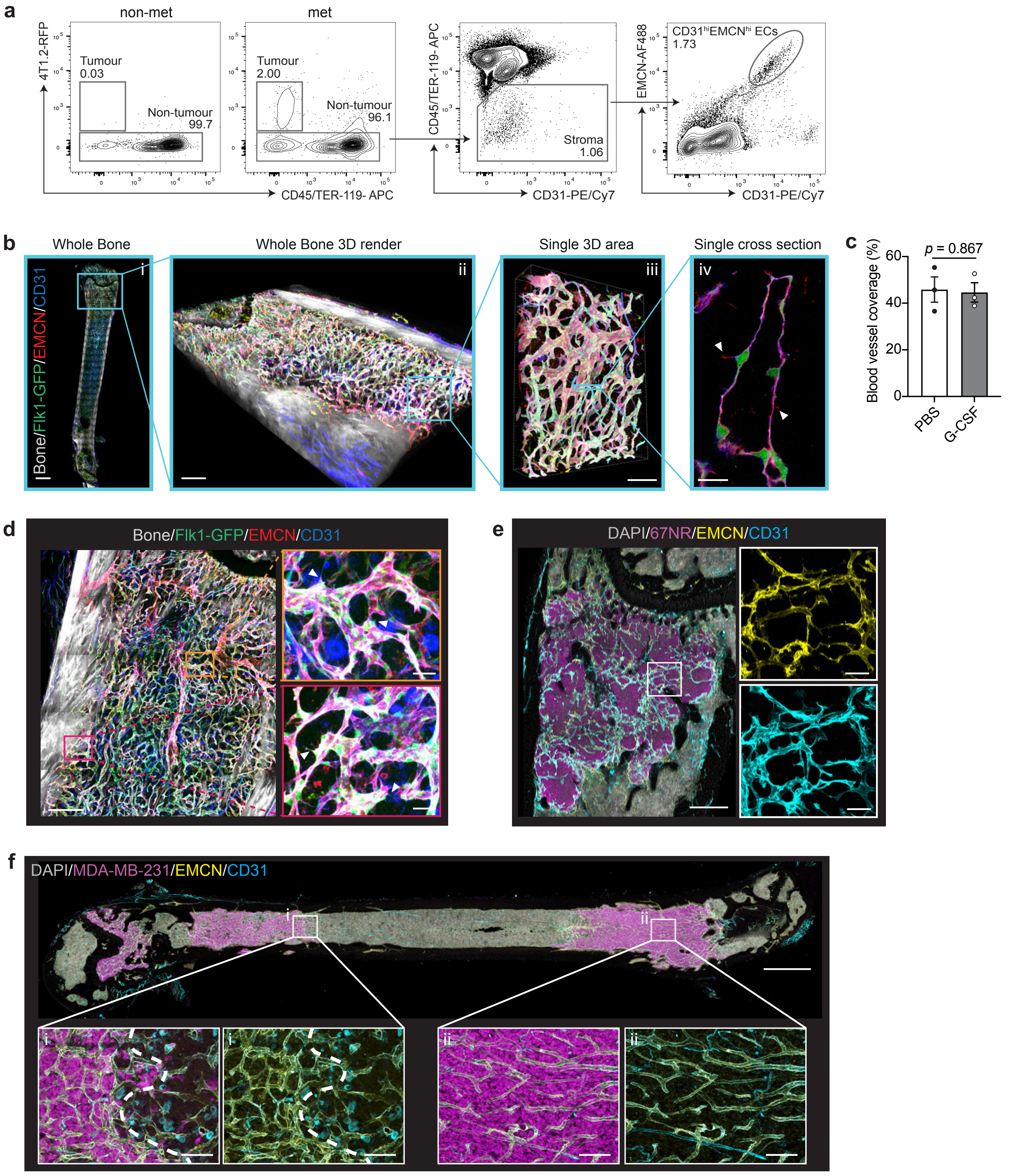

Supplementary Figure 3

Supplementary Fig. 3: G-CSF drives vessel remodelling in BM. a, FACS plots showing gating strategy for $CD31^{hi}EMCN^{hi}$ ECs from tumour-free (non-met) and adjacent metastasis-infiltrated (met) areas of the same bone. Numbers indicate the percentage of cells within gates. Doublets and DAPI-positive dead cells were excluded. **b**, Examples of 3D multiphoton images acquired at different magnifications, from whole bone to subcellular details of a *Flk1-GFP* (green) femoral BM slice immunostained for EMCN (red) and CD31 (blue). Bone collagen was defined by second harmonic signal (grey). See also Supplementary Movie 3. Scale bars: 1 mm (i), 200 µm (ii), 100 µm (iii), 50 µm (iv). **c**, Percentages of bone marrow (BM) covered by blood vessels in PBS and G-CSF treated *Flk1-GFP* mice. *n*=3 mice per group. *P* value, two-tailed paired *t*-test. **d**, Left: 3D image of femoral BM of *Flk1-GFP* mouse following G-CSF treatment immunostained for EMCN (red) and CD31 (blue). Bone collagen was defined by second harmonic signal (grey). Right: enlarged images of sprouting vasculature in central BM (marked by orange border) and endosteum (marked by red border) (*n*=3 mice). Arrowheads indicate endothelial sprouts. Scale bars: 200 µm (overview), 30 µm (enlargements). **e**, 3D confocal image of a 67NR lesion (magenta) in femoral BM stained for DAPI (grey), EMCN (yellow) and CD31 (cyan) (*n*=5 mice). Scale bars: 300 µm (overview), 50 µm (enlargements). **f**, 3D images of MDA-MB-231 metastases (magenta) in femoral BM stained for DAPI (grey), EMCN (yellow) and CD31 (cyan). Bottom panels show enlargements from the selected regions in top panel. Dotted lines mark the lesion boundary (*n*=3 mice). Scale bar: 1 mm (overview), 100 µm (enlargement). All data reflect mean ± s.e.m. Source data are provided as a Source Data file.

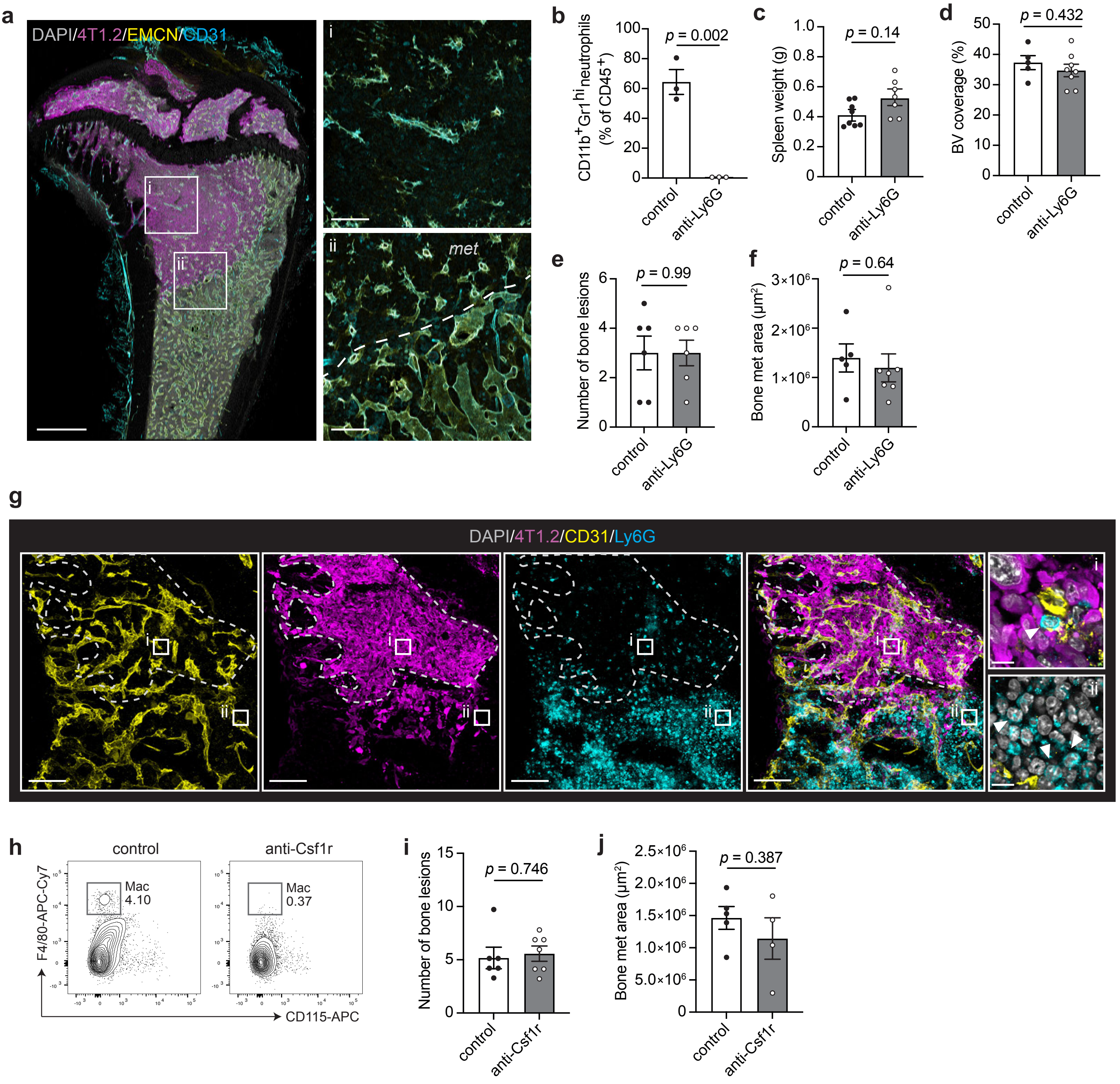

Supplementary Figure 4

Supplementary Fig. 4: Ablation of B and T lymphoid cells, NK cells, neutrophils and macrophages does not affect metastases-driven vessel remodelling and lesion outgrowth. **a**, 3D images of 4T1.2 macrometastases (met; magenta) in the bone marrow (BM) of a NSG mouse, stained for DAPI (grey), EMCN (yellow) and CD31 (cyan) (*n*=2 mice). Dashed line marks the lesion boundary. Scale bars, 500 µm (overview), 100 µm (enlargements). **b**, **c**, FACS analysis of BM neutrophils (**b**) and spleen weight (**c**) of metastasis-bearing mice treated with isotype control or anti-Ly6G antibody (Ab) (b). For **b**, *n*=3 mice per group. For **c**, *n*=8 (control) and 7 mice (anti-Ly6G). *P* values, two-tailed unpaired *t*-tests. **d-f**, Image-based measurement of blood vessel coverage within lesion (**d**), number of lesions (**e**), and lesion area (**f**) in bones collected from metastasis-bearing mice treated with isotype control or anti-Ly6G Ab. For **d**, **f**, *n*=5 mice per group. For **e**, *n*=6 mice per group. *P* values, two-tailed unpaired *t*-tests. **g**, 3D images of 4T1.2 bone metastasis (magenta) with enlarged optical sections of the metastatic lesion (i) and adjacent tumour-free area (ii), stained for DAPI (grey), CD31 (yellow) and Ly6G (cyan) (*n*=3 mice). Dotted lines demarcate lesion area. Arrowheads indicate $Ly6G^+$ neutrophils. Scale bars, 50 µm (overview), 10 µm (enlargement). **h**, FACS plots of BM macrophages (Mac) from isotype control or anti-Csf1r Ab-treated metastasis-bearing mice. Numbers indicate the percentage of cells within gates. Doublets and DAPI-positive dead cells were excluded. **i**, **j**, Image-based quantification of number of lesions (**i**) and lesion area (**j**) in bones collected from metastasis-bearing mice treated with isotype control or anti-Csf1r Ab. For **i**, *n*=6 (control) and 7 mice (anti-Csf1r). For **j**, *n*=5 (control) and 4 mice (anti-Csf1r). *P* values, two-tailed unpaired *t*-tests. All data represent mean ± s.e.m. Source data are provided as a Source Data file.

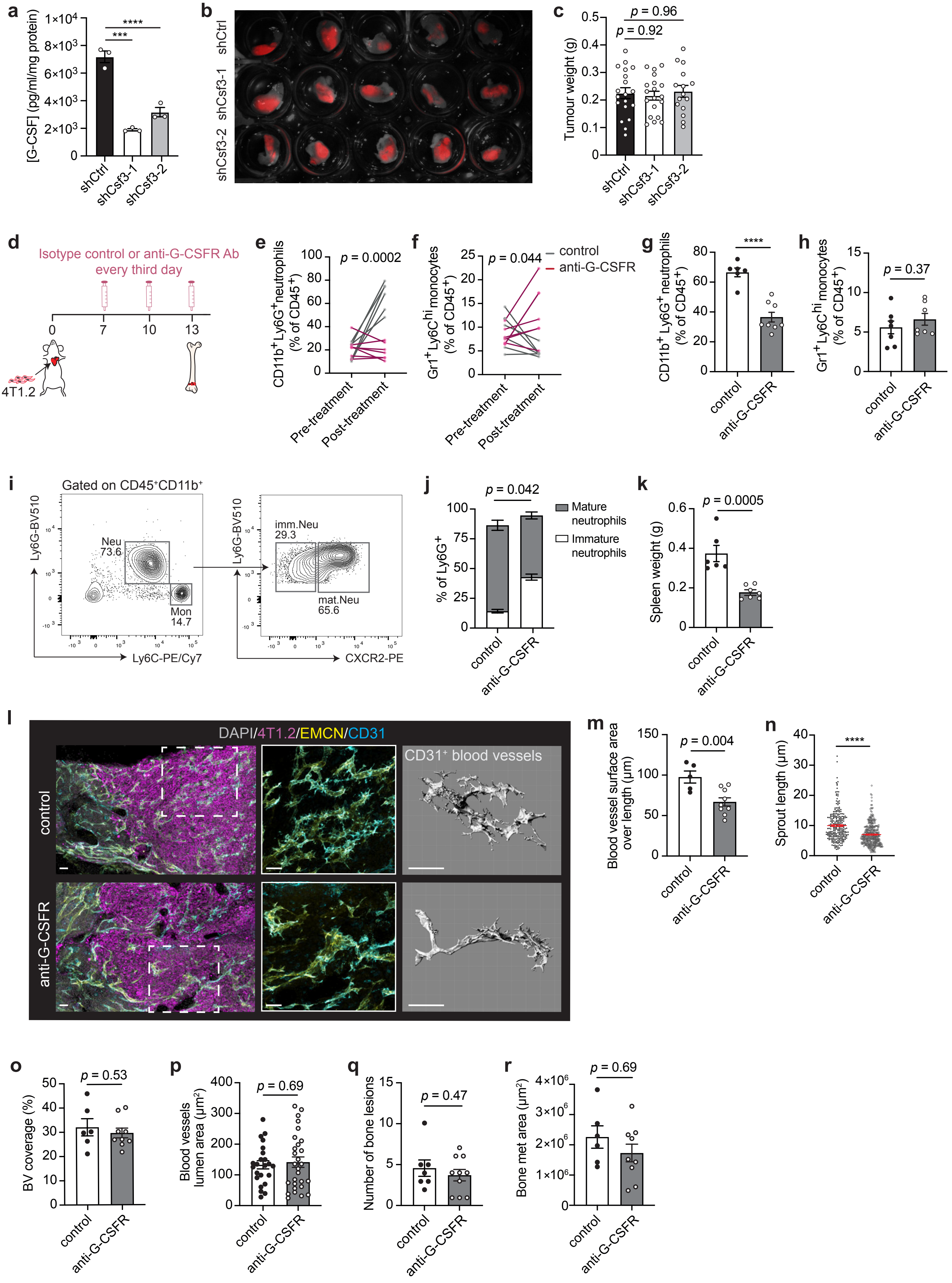

Supplementary Figure 5

Supplementary Fig. 5: Effect of genetic or pharmacological ablation of G-CSF on primary tumorigenesis and distant metastasis. a, ELISA for G-CSF in cell media of the indicated cell lines. *n*=3 per cell line. ***$P = 0.002$, ****$P < 0.0001$. **b**, **c**, Ex vivo fluorescence imaging (**b**) and weight (**c**) of primary tumour formed by the indicated cell lines. *n*=19 (shCtrl, shCsf3-1) and 16 mice (shCsf3-2). **d**, Diagram showing the experimental plan in **e**-**r**. **e**, **f**, FACS analysis of circulating neutrophils (**e**) and monocytes (**f**) before and after treatment. *n*=5 (**e**), 6 (**f**) mice for control; *n*=6 (**e**), 7 (**f**) mice for anti-G-CSFR. **g**-**k**, FACS analysis of BM neutrophils (Neu) (**g**), monocytes (Mon) (**h**) and immature (imm.Neu) and mature neutrophils (mat.Neu) (**j**), and spleen weight (**k**) at experimental endpoint. Representative FACS plots are provided in **i**. *n*=6 (**g**), 7 (**h**), 3 (**j**), 6 (**k**) mice for control; *n*=8 (**g**), 7 (**h**), 3 (**j**), 7 (**k**) mice for anti-G-CSFR. ****$P < 0.0001$. **l**, Tile scan (left), magnified images (middle), and digitally reconstructed $CD31^+$ blood vessels (right) in bone lesions of mice treated with isotype control or anti-G-CSFR Ab. Bones were stained for DAPI (grey), 4T1.2 cells (magenta), EMCN (yellow) and CD31 (cyan) (*n*=6 mice per group). Scale bars: 50 µm. **m**, **n,** Quantification of surface area over length (**m**), and sprout length (**n**) of blood vessels from **l**. Individual dots are from one vessel (**m**) or sprout (**n**). *n*=5 (**m**), 3 (**n**) mice for control; *n*=6 (**m**), 3 (**n**) mice for anti-G-CSFR. ****$P < 0.0001$. **o**-**r**, Quantification of density (**o**) and lumen area (**p**) of vessels at bone lesions, and number (**q**) and area (**r**) of lesions from **l**. *n*=5 (**o**), 3 (**p**), 7 (**q**), 4 (**r**) mice for control; *n*=7 (**o**), 4 (**p**), 10 (**q**), 8 (**r**) mice for anti-G-CSFR. *P* values in **a**, **c** by one-way ANOVA and Dunnett's multiple comparisons test, in **e**, **f** by repeated measure two-way ANOVA, in **j** by two-way ANOVA, and in **g**, **h**, **k**, **m**-**r** by two-tailed unpaired *t*-tests. All data represent mean ± s.e.m. Source data are provided as a Source Data file.

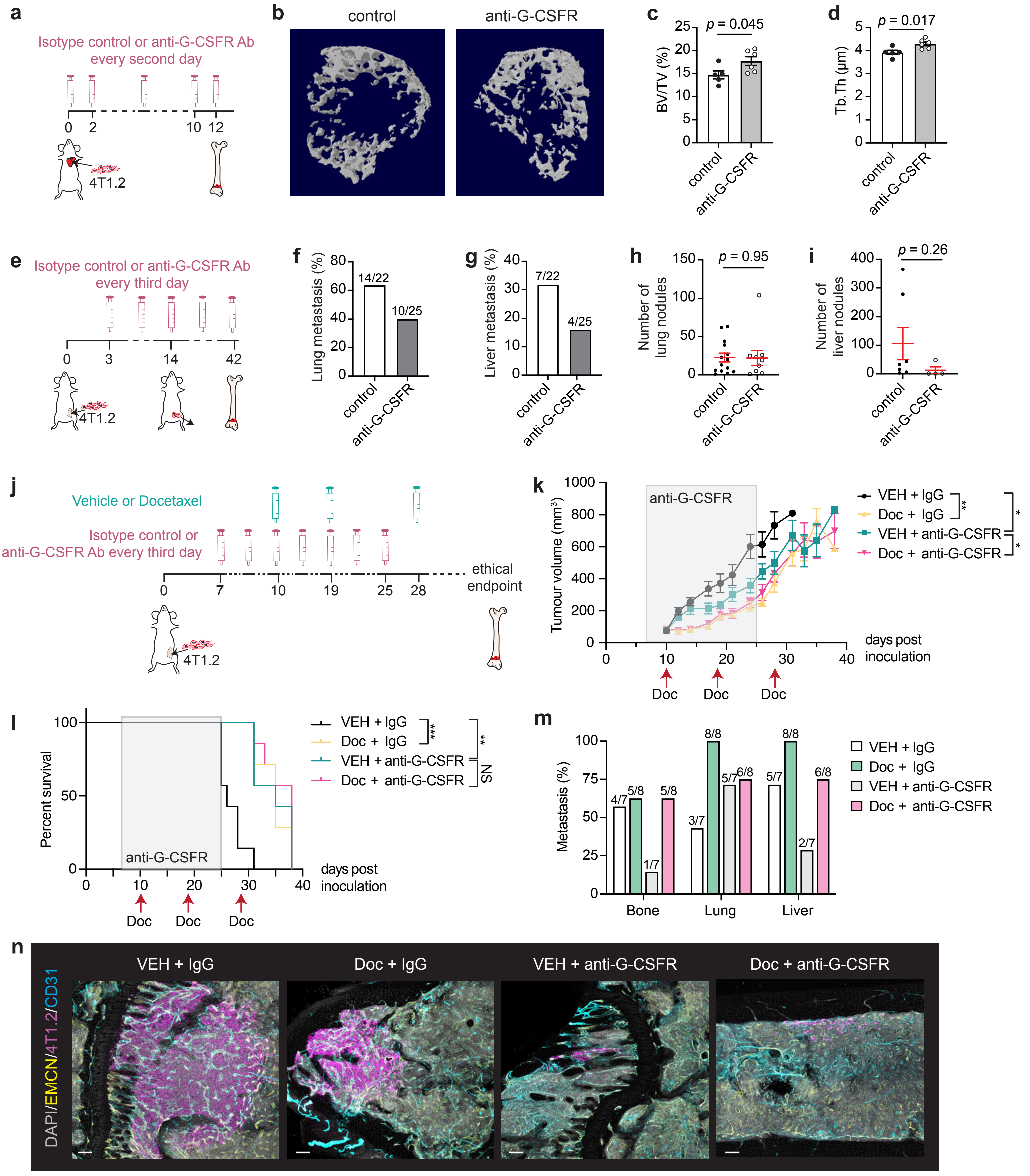

Supplementary Figure 6

Supplementary Fig. 6: Assessment of the anti-metastatic effect of anti-G-CSFR neutralising antibody and docetaxel in experimental and spontaneous bone metastasis models. **a**, Diagram showing the experimental plan in **b**-**d**. **b**-**d**, Representative micro-computed tomography images (**b**) and morphometric analysis of bone volume/tissue volume (BV/TV; %) (**c**) and trabecular thickness (Tb.Th; μm) (**d**) of tibia from metastasis-bearing mice. *n*=5 (control) and 6 mice (anti-G-CSFR). *P* values, two-tailed unpaired *t*-test. **e**, Diagram showing the experimental plan in **f**-**i**. **f**-**i**, Incidence of lung (**f**) and liver (**g**) metastasis, and number of metastatic nodules in lung (**h**) and liver (**i**) in control (*n*=22 mice) or anti-G-CSFR (*n*=25 mice) groups at experimental endpoint. *P* values, two-tailed unpaired *t*-test. **j**, Diagram showing the experimental plan in **k**-**n**. **k**, Orthotopic tumour growth in each group. *n*=7 mice for VEH + IgG and VEH + anti-G-CSFR Ab, *n*=8 mice for Doc + IgG and Doc + anti-G-CSFR Ab group. *P = 0.01, **P = 0.002 by two-way repeated measures ANOVA (Day 0 to 14). **l**, Kaplan-Meier survival analysis of mice in each group. *n*=7 mice per group. **P = 0.0038, ***P = 0.0006 by two-sided log-rank Mantel-Cox test and two-sided Gehan-Breslow-Wilcoxon method. NS, not significant. **m**, Incidence of metastasis in bone, lung and liver in each group at ethical endpoint . *n*=7 mice for VEH + IgG and VEH + anti-G-CSFR Ab, *n*=8 mice for Doc + IgG and Doc + anti-G-CSFR Ab. **n**, 3D images of bone marrow from mice in each group at ethical endpoint. Bones were stained for DAPI (grey), 4T1.2 cells (magenta), EMCN (yellow) and CD31 (cyan) (n=3 mice per group, except *n*=1 mouse for VEH+ anti-G-CSFR). Scale bars: 200 μm. All data represent mean ± s.e.m. Source data are provided as a Source Data file.
