# Supplementary material for: Mammary tumour cells remodel the bone marrow vascular microenvironment to support metastasis
Source: Nat Commun. 2021 Nov 26;12:6920. doi: 10.1038/s41467-021-26556-6 (PMC8626461; doi:10.1038/s41467-021-26556-6)
Supplement: Supplementary file 2 — Description of Additional Supplementary Files [file 41467_2021_26556_MOESM2_ESM.pdf]

Description of Additional Supplementary Files

File Name: Supplementary Movie 1
Description: Confocal deep imaging of BM vasculature immunolabelled with anti-CD31 (cyan). The movie cycles through optical sections of the marrow at the xy plane over a depth of 184 µm at a step size of 1.21 µm. Optical clearing allows deep tissue imaging with minimal loss of signal intensity. The intact lumen and continuity of vessels confirm the preservation of tissue microarchitecture.

File Name: Supplementary Movie 2
Description: 3D reconstruction of the bone metastatic microenvironment immunolabelled with anti-RFP and anti-CD31 antibodies to mark 4T1.2 tumour cells (yellow) and the vasculature (grey), respectively. Arrows point to tumour cells that are intimately associated with BM endothelium.

File Name: Supplementary Movie 3
Description: Multiphoton confocal imaging of a femoral bone from a *Flk1-GFP* mouse at varying magnifications and resolutions. Initial whole-bone scanning provides a general overview of the vascular network throughout the entire tissue. Regions of interest are then subsequently captured at high resolution that permits precise visualisation and quantification of subcellular details.

File Name: Supplementary Movie 4
Description: Representative 3D image and scanning of *xz* and *xy* optical sections of femoral bone from an untreated *Flk1-GFP* mouse. The specimen was immunostained for EMCN (yellow) and CD31 (magenta). Endogenous *Flk1-GFP* signal shows endothelial cell nuclei.

File Name: Supplementary Movie 5
Description: Representative 3D image and scanning of *xz* and *xy* optical sections of a femoral bone from a *Flk1-GFP* mouse treated with Filgrastim (recombinant human G-CSF). The specimen was immunostained for EMCN (yellow) and CD31 (magenta). Endogenous *Flk1-GFP* signal labels endothelial cell nuclei, as well as numerous EMCN+CD31+ cytoplasmic projections.
